# Hypoxic stress is an early pathogenic event in human *VCP*-mutant ALS astrocytes

**DOI:** 10.1016/j.stemcr.2025.102723

**Published:** 2025-12-04

**Authors:** Hannah D. Franklin, Hamish Crerar, Nishita Parnandi, Michael Lattke, Stanislaw Majewski, Benjamin E. Clarke, Husayn Pallikonda, Michael Howell, Simon J. Boulton, Rickie Patani

**Affiliations:** 1 The Francis Crick Institute, 1 Midland Road, London NW1 1AT, UK; 2 Department of Molecular Neuroscience, UCL Institute of Neurology, Queen Square, London, UK; 3 Department of Brain Sciences, Imperial College London, Hammersmith Hospital Campus, Du Cane Road, London W12 0NN, UK; 4 Neurobiology Programme, Life Sciences Institute, Centre for Life Sciences, National University of Singapore, Singapore, Singapore; 5 Department of Medicine, Yong Loo Lin School of Medicine, National University of Singapore, Singapore, Singapore; 6 Department of Anatomy, Yong Loo Lin School of Medicine, National University of Singapore, Singapore, Singapore

**Keywords:** hypoxia, amyotrophic lateral sclerosis, astrocytes, VCP mutation, disease modelling, human iPSCs, HIF-1α, neurodegeneration, RNA-binding proteins

## Abstract

Astrocytes are essential regulators of neuronal health, and their dysfunction contributes to neurodegenerative diseases such as amyotrophic lateral sclerosis (ALS). Using human induced pluripotent stem cell (iPSC)-derived astrocytes carrying ALS-associated *VCP* mutations, we uncover cell-autonomous activation of the hypoxia response under basal conditions. *VCP*-mutant astrocytes exhibit increased nuclear hypoxia-inducible factor (HIF)-1ɑ, mitochondrial depolarization, and lipid droplet accumulation. Mimicking hypoxia in control astrocytes by HIF-1ɑ stabilization with dimethyloxalylglycine recapitulates these phenotypes. Transcriptomic and CUT&RUN profiling reveal direct HIF-1ɑ binding to canonical hypoxia-responsive genes in *VCP*-mutant astrocytes and a transcriptional signature of metabolic reprogramming and mitochondrial dysfunction under normoxia. Furthermore, conditioned medium from hypoxia-exposed astrocytes fails to rescue RNA-binding protein mislocalization in motor neurons, unlike medium from healthy counterparts. Together, these findings demonstrate that aberrant HIF-1ɑ activation drives astrocytic dysfunction and compromises neuronal support, identifying hypoxic stress as an early and functionally consequential event in *VCP*-mutant ALS, with therapeutic implications for targeting HIF-1ɑ signaling.

## Highlights

• *VCP*-ALS astrocytes show cell-autonomous HIF-1α activation at baseline
• Hypoxia or DMOG alone recapitulate mitochondrial and lipid droplet phenotypes
• CUT&RUN confirms HIF-1α binding to canonical targets in *VCP*-ALS astrocytes
• Hypoxic astrocytes fail to correct *VCP*-ALS neuronal RBP mislocalization

## Teaser

In this article, Franklin and colleagues show that human *VCP*-ALS astrocytes exhibit hypoxic stress through basal HIF-1α activation and binding to canonical targets, mitochondrial depolarization, and lipid droplet accumulation. Hypoxia or DMOG exposure phenocopies these defects in control astrocytes, which then fail to correct *VCP*-ALS motor neuron RNA-binding protein mislocalization. HIF-1α signaling is a potential therapeutic target in ALS.

## Introduction

Oxygen homeostasis is vital for cellular survival, particularly in the brain, which is highly susceptible to hypoxia (Sharp and Bernaudin 2004). Insufficient oxygen delivery and/or increased metabolic demands cause hypoxia, which triggers various cellular responses depending on its severity and duration. While prolonged or severe hypoxia can lead to neuronal dysfunction and subsequent degeneration, transient hypoxia can activate protective homeostatic mechanisms (Rodriguez et al., 2021; Chen et al., 2022).

Astrocytes abound in the central nervous system (CNS) and are increasingly recognized for their role in neurodegenerative diseases, including amyotrophic lateral sclerosis (ALS) (Franklin et al. 2021; Guttenplan et al., 2020; Stoklund Dittlau et al., 2023; Birger et al., 2019; Nagai et al., 2007; Di Giorgio et al., 2008). Beyond their traditional supportive roles, astrocytes are key regulators of cerebral oxygen and energy metabolism. They modulate neurovascular coupling to ensure appropriate oxygen and nutrient delivery to active brain regions, and they play a pivotal role in coordinating metabolic flux between blood vessels and neurons through processes such as the astrocyte-neuron lactate shuttle (Attwell et al., 2010; Bélanger et al. 2011).

Although hypoxia has been widely accepted as a common feature of neurodegeneration, the molecular processes induced by hypoxia in the CNS have primarily been explored in the context of stroke, spinal cord injury, and the neuronal subtypes most affected by these conditions. However, our recent meta-analysis of a myriad of ALS astrocyte transcriptomic datasets has identified hypoxia as one of the most significantly activated pathway in these cells (Ziff et al., 2022). This result was surprising as earlier research suggested that an ischemia-reperfusion paradigm in rodent models is sufficient to induce a *neuroprotective* state in astrocytes (Zamanian et al., 2012). This apparent controversy—where hypoxia is associated with both neuroprotective and neurodegenerative responses—underscores the necessity for further investigation into the precise role of hypoxic stress in ALS astrocytes.

Central to the cellular response to hypoxia is the hypoxia-inducible factor (HIF) pathway. HIFs, especially HIF-1α, play a critical role in cellular adaptation to hypoxia by modulating the expression of genes involved in processes like angiogenesis, glycolysis, mitochondrial function, and cell survival (Semenza and Wang 1992; Wang et al., 1995; Semenza 2000). Under normoxic conditions, HIF-1α is rapidly degraded, but during hypoxia, it stabilizes and translocates to the nucleus, where it dimerizes with its β-subunit counterpart and forms an active complex that modulates the expression of target genes by binding to the hypoxia response element (HRE) in their promoter regions. While transient activation of HIF-1α is typically protective, prolonged activation is linked to neurodegeneration, highlighting the salience of precise and temporally coordinated cellular adaptation to hypoxic stress. Emerging evidence suggests an important role for hypoxia responses in neurodegenerative diseases, including ALS. Indeed, mutations in a hypoxia response gene *ANG* have been associated with ALS (Conforti et al., 2008; Greenway et al., 2004; Sebastià et al., 2009). Maladaptive hypoxia responses, including aberrant HIF-1α activity, have also been observed in ALS (Moreau et al., 2011; Just et al., 2007). Notably, deletion of the HRE in the VEGF promoter region has been linked to motor neuron (MN) degeneration (Oosthuyse et al., 2001), and therapeutic strategies targeting VEGF in ALS models have shown neuroprotective effects (Azzouz et al., 2004). However, the contribution of glial cells, particularly astrocytes, to these hypoxia-related mechanisms remains incompletely understood.

In parallel, ALS pathogenesis is tightly linked to the dysfunction of an increasing number of RNA-binding proteins (RBPs), including FUS and SFPQ, which regulate splicing, RNA transport, and stability. Nuclear-to-cytoplasmic (N:C) mislocalization of these RBPs is a hallmark of ALS MNs, contributing to RNA processing defects and neuronal vulnerability. Notably, FUS and SFPQ mislocalization has been observed not only in sporadic ALS but also in *VCP*-mutant models, implicating their dysregulation as a convergent feature of the disease (Luisier et al., 2018; Tyzack et al., 2019).

Astrocytes have been shown to influence neuronal RBP pathology in a non-cell-autonomous manner; notably, healthy astrocytes can mitigate TDP-43 mislocalization, aggregation and toxicity (Smethurst et al., 2020). Importantly, hypoxia itself has been reported to affect RBP dynamics (Ho et al., 2020; Masuda et al. 2009). In astrocytes, the RBP HuR translocates to the cytoplasm during hypoxic stress, where it modulates both HIF-1ɑ translation and cytokine expression (Kwan et al., 2017), and has also been shown to positively regulate the expression of other ALS-linked RBPs, including TDP-43 and FUS (Lu et al., 2014). These findings raise the possibility that astrocytic hypoxic stress may not only drive intrinsic metabolic dysfunction but also influence RBP localization in neighboring MNs.

To address these intersecting knowledge gaps—how hypoxia pathway activation contributes to astrocyte dysfunction and how this in turn may influence neuronal RBP pathology—we harnessed our established method for generating astrocytes from human induced pluripotent stem cells (hiPSCs), where we have previously shown ALS-relevant cell-autonomous phenotypes (Tyzack et al., 2017; Taha et al., 2022; Ziff et al., 2021; Hall et al., 2017). This model enables the dissection of both intrinsic astrocytic mechanisms and their non-cell-autonomous effects on MNs, providing an ideal platform to investigate how hypoxic stress and HIF-1ɑ dysregulation contribute to ALS pathogenesis.

In the current study, we show that *VCP*-mutant astrocytes exhibit intrinsic activation of the hypoxia pathway, characterized by mitochondrial dysfunction and lipid droplet (LD) accumulation. We demonstrate that exposure of control astrocytes to hypoxia is sufficient to recapitulate these phenotypes and that they are HIF-1ɑ dependent. Using RNA sequencing (RNA-seq) and CUT&RUN, we further show that nuclear HIF-1ɑ in basal *VCP*-mutant astrocytes binds to canonical hypoxia response genes and that these are differentially expressed, mimicking control astrocytes subjected to a hypoxic stimulus. Finally, we extend these findings to a neuron-glial communication context, showing that hypoxia-exposed astrocytes lose their ability to non-cell-autonomously correct RBP mislocalization in MNs—linking astrocytic hypoxic stress to a canonical hallmark of ALS. Taken together, our findings establish that hypoxic stress is an early pathogenic event in *VCP*-mutant ALS astrocytes.

## Results

### hiPSC-derived *VCP*-mutant ALS astrocytes display mitochondrial dysfunction, lipid droplet accumulation, and increased HIF-1ɑ nuclear translocation

**Figure 1:**
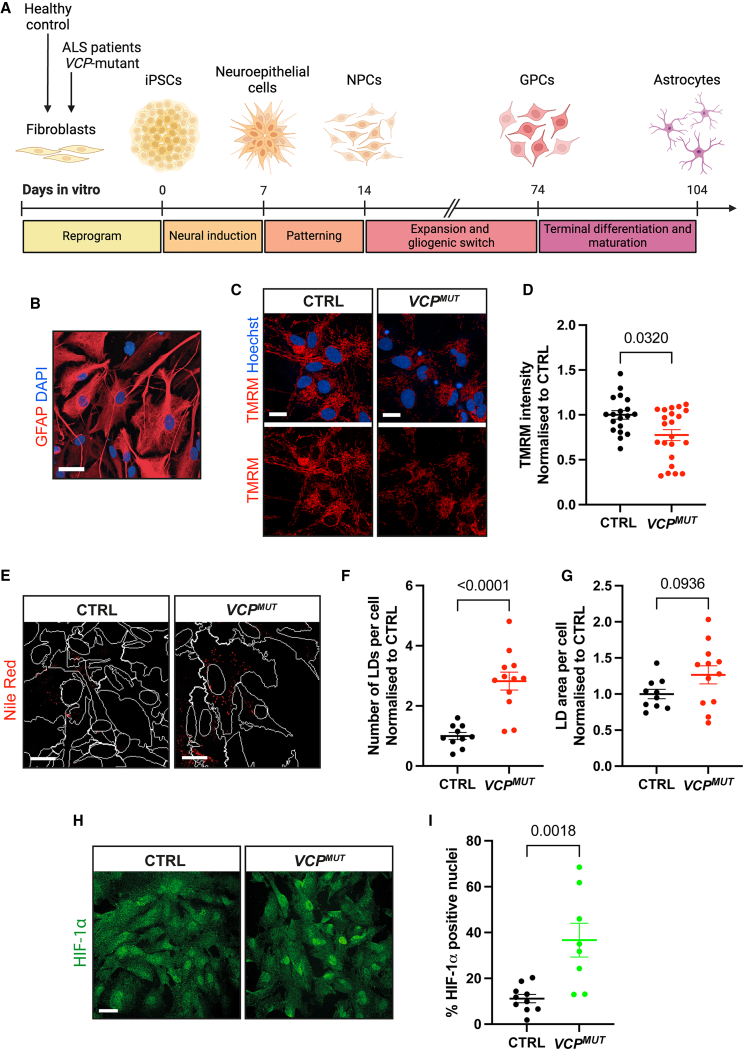
hiPSC-derived *VCP*-mutant ALS astrocytes display mitochondrial dysfunction, LD accumulation, and increased HIF-1ɑ nuclear translocation (A) Schematic illustration of directed differentiation paradigm for generation of highly enriched astrocytes from hiPSCs derived from healthy control (CTRL) and *VCP*-mutant (*VCP*^*MUT*^) ALS patient fibroblasts. (B) Representative fluorescence image of differentiated CTRL hiPSC-derived astrocytes, immunolabeled with astrocyte marker glial fibrillary acidic protein (GFAP) (red) and stained with DAPI (blue). Scale bar, 30 μm. (C) Representative live-cell fluorescence images of CTRL and *VCP*^*MUT*^ astrocytes stained with TMRM (red) to visualize mitochondrial membrane potential and Hoechst (blue). Scale bars, 20 μm. (D) Quantification of TMRM intensity, normalized to CTRL within each experimental repeat (cell lines used in Repeat 1: CTRL2, CTRL4, CTRL6, NCRM E6, Mut1.1, Mut1.2, and Mut2.2; Repeat 2: CTRL3, CTRL5, CTRL6, Mut1.2, and Mut2.1; Repeat 3: CTRL1, CTRL2, CTRL3, CTRL6, NCRM C2, NCRM E6, Mut1.1, Mut1.2, Mut2.1, and Mut2.2). *p* value calculated from Mann-Whitney test. (E) Representative fluorescence images of CTRL and *VCP*^*MUT*^ astrocytes stained with Nile Red (red) to visualize LD accumulation. LDs appear as puncta emitting red fluorescence. Nuclear (DAPI) and cytoplasmic (GFAP) masks are marked by white traces. Scale bars, 20 μm. (F–G) Quantification of (F) the number of Nile Red-stained LDs per cell and (G) LD area (pixels) per cell. Each data point represents the mean value of 10 fields per technical repeat per cell line (2 technical repeats per condition). Data normalized to CTRL per experimental repeat (cell lines used in Repeat 1: CTRL1, CTRL2, CTRL6, NCRM C2, NCRM E6, Mut1.1, and Mut2.1; Repeat 2: CTRL5, CTRL6, Mut1.2, and Mut2.1). *p* values calculated from unpaired *t* test. (H) Representative fluorescence images of CTRL and *VCP*^*MUT*^ astrocytes immunolabeled with HIF-1ɑ (green). Scale bar, 20 μm. (I) Scatterplot depicting quantitative immunocytochemistry cell-by-cell analysis of the % of nuclei exhibiting cytoplasmic-to-nuclear translocation of HIF-1ɑ. Data are representative of 10 fields acquired per technical repeat, 2 technical repeats per cell line (cell lines used in Repeat 1: CTRL1, CTRL4, VCPF10, and Mut 2.1; Repeat 2: CTRL1, CTRL4, Mut 1.1, and Mut2.1; Repeat 3: CTRL4, VCPF10, and Mut2.2). *p* values calculated from unpaired *t* test. All data points in scatter dot plots represent the mean value per technical replicate, and error bars represent mean ± SEM.

Our previous meta-analysis of hiPSC-derived ALS astrocyte datasets revealed hypoxia response as one of the most significantly activated signaling pathways (Ziff et al., 2022). To investigate the functional relevance of this finding, we utilized our established and validated protocol for differentiating astrocytes from hiPSCs (Hall et al., 2017; Tyzack et al., 2017; Ziff et al., 2021) (Figures 1A and 1B) with a modest adaptation in the terminal differentiation phase (see methods for details). We compared astrocytes derived from control individuals (CTRL) with those carrying ALS-causing mutations in the *VCP* gene (*VCP*^*MUT*^), a model previously shown to capture ALS-relevant astrocyte phenotypes (Taha et al., 2022; Ziff et al., 2022).

Given the known impact of hypoxia on mitochondrial activity, and of mitochondrial dysfunction in neurodegeneration, we first sought to determine if our cell types displayed mitochondrial phenotypes. Assessment of mitochondrial function by Tetramethylrhodamine methyl ester (TMRM) staining revealed significant mitochondrial membrane depolarization in *VCP*^*MUT*^ astrocytes compared to their CTRL counterparts (Figures 1C and 1D). Mitochondrial morphology was examined using MitoTracker staining, which revealed a non-significant trend toward reduced mitochondrial area in *VCP*^*MUT*^ astrocytes compared with CTRLs (Figure S1), suggestive of possible mitochondrial fragmentation, decreased mitochondrial number, or reduced mitochondrial mass.

Given the link between mitochondrial dysfunction and lipid metabolism, we next assessed LD phenotypes using Nile Red staining. *VCP*^*MUT*^ astrocytes exhibited a significantly higher number of LDs (Figures 1E and 1F) and a trend toward increased LD area (Figure 1G), suggesting altered lipid homeostasis. To determine whether the hypoxia pathway was activated at the protein level, noting that the meta-analysis of ALS astrocytes was only at the transcript level, we assessed HIF-1ɑ localization by immunofluorescence. *VCP*^*MUT*^ astrocytes showed significantly increased nuclear HIF-1ɑ accumulation compared to CTRLs (Figures 1H and 1I), consistent with hypoxia pathway activation. Collectively, these findings demonstrate that hiPSC-derived *VCP*^*MUT*^ ALS astrocytes exhibit mitochondrial and metabolic abnormalities, accompanied by cell-autonomous activation of the hypoxia response.

### Establishing optimized hypoxic stress assays in hiPSC astrocytes

To elucidate the relationship between increased HIF-1ɑ nuclear translocation, activation of hypoxia pathways, and cell-autonomous astrocyte phenotypes in the context of ALS, we established an efficient hypoxia paradigm *in vitro*. We employed two methods: (1) a controlled oxygen environment (1% for 24 h; Figure S2A) using InvivO_2_ and SCI-tive hypoxia workstations (Baker Ruskinn) and (2) a pharmacological approach using dimethyloxalylglycine (DMOG), a HIF-1ɑ stabilizer that inhibits prolyl hydroxylase domain enzyme, which ordinarily targets HIF-1ɑ for degradation in the absence of hypoxia; thus DMOG causes HIF-1ɑ translocation to the nucleus under normoxic conditions (Figure S2A is a schematic representation of this part of our study). Comparison of these methods by western blot analysis of HIF-1ɑ protein levels in two hiPSC-derived CTRL astrocyte lines revealed that DMOG treatment resulted in a greater HIF-1ɑ accumulation compared to 1% O_2_ exposure (Figures S2B and S2C). Quantitative immunofluorescence demonstrated that HIF-1ɑ translocates to the nucleus following a 24-h exposure to 1% O_2_ in both CTRL and *VCP*^*MUT*^ astrocytes (Figures S2D and S2E). DMOG treatment stabilized nuclear HIF-1ɑ in hiPSC-derived astrocytes under basal conditions at least as effectively as 1% O_2_ exposure (Figures S2F and S2G). Quantitative reverse-transcription PCR analysis confirmed increased expression of key HIF-1ɑ target genes (*PDK1*, *ANG* and *VEGF*) following hypoxia exposure, while *HIF-1ɑ* transcript levels remained unchanged (Figure S2H). Notably, gene expression changes following hypoxia exposure only revealed significance for *VEGF* in *VCP*^*MUT*^ astrocytes, but not for *PDK1* and *ANG*, possibly reflecting their pre-existing state of hypoxic activation or compromised HIF-1ɑ reactivity.

### *VCP*-mutant astrocytes exhibit altered transcriptional responses to hypoxia

To further interrogate the transcriptional landscape caused by hypoxia pathway activation in *VCP*^*MUT*^ astrocytes, we performed RNA-seq of hiPSC-derived astrocytes under both normoxic and hypoxic conditions. We included four experimental conditions: CTRL astrocytes under basal conditions/normoxia, CTRL astrocytes after 24-h hypoxia, *VCP*^*MUT*^ astrocytes under basal conditions/normoxia, and *VCP*^*MUT*^ astrocytes after 24-h hypoxia. Each group was profiled using PolyA-selected RNA-seq (∼50 million reads per sample).

We first confirmed that astrocyte identity/maturation was comparable across genotypes and oxygen conditions. Using human orthologs of established mouse astrocyte maturation genes (Lattke et al., 2021), bulk RNA-seq showed no genotype- or hypoxia-dependent differences in maturation marker expression (Figure S3A). To characterize the sources of variation in the dataset in an unbiased manner, we performed principal-component (PC) analysis. Noting the different genetic background of the original iPSC lines, the first two PCs explaining the highest proportion of transcriptional variation (PC1/2) separated cell-lines independent of *VCP* genotype or hypoxia treatment (Figure S3B). PC3 separated *VCP*^*MUT*^ from CTRL samples, while PC4 separated hypoxia vs. normoxia samples (Figure S3C), indicating major independent effects of *VCP* genotype or hypoxia treatment on the global transcriptome. Interestingly, PC5 separated normoxic CTRL samples from both *VCP*^*MUT*^ and hypoxic CTRL samples (Figure S3D), indicating shared changes between *VCP*^*MUT*^ and hypoxic CTRL samples compared to normoxic controls impacting the global transcriptomic state.

**Figure 2:**
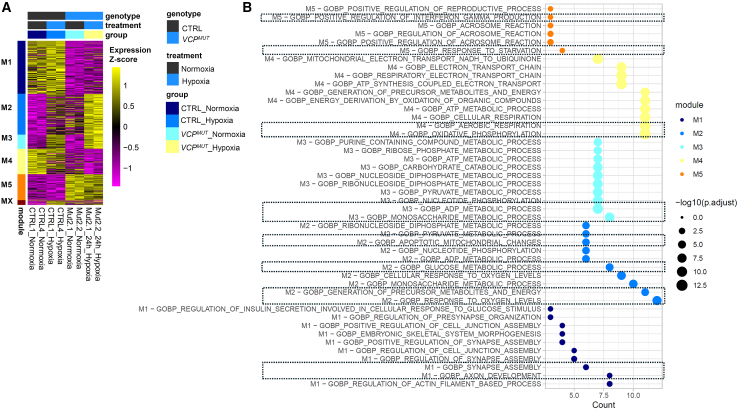
*VCP*-mutant ALS astrocytes display an altered transcriptional hypoxia response (A) Heatmap shows relative expression of genes affected by 24-h hypoxia (vs. normoxia) and/or the *VCP* mutation (*VCP*^*MUT*^) vs. control (CTRL), grouped into co-expressed modules M1–M5 (MX: genes not linked to any co-expression pattern). Cell lines: CTRL1, CTRL4, Mut2.1, and Mut2.2. (B) Top 10 enriched GO terms from MSigDB database for each co-expression module, number of differential genes in GO term, and FDR for enrichment. Highlighted: selected functions potentially relevant to ALS pathogenesis.

To identify genes affected by the four experimental conditions, we performed differential gene expression analysis, which identified 417 genes significantly regulated by genotype, hypoxia, or their interaction (false discovery rate [FDR] ≤0.05). These genes were grouped into five co-expression modules (M1–M5) using unsupervised clustering (see methods; Figure 2A). Each module exhibited a distinct expression pattern and biological signature.

Activity of module M1 (135 genes) was strongly reduced in *VCP*^*MUT*^ astrocytes compared to controls, but only minimally affected by hypoxia, and was enriched for genes linked to neuronal maturation, like *GABRA2*, *SLITRK2*, *CBLN2*, *SLITRK4*, *EPHA5*, *EPHA7*, *KLF4*, and *NRXN3*, indicated by enrichment for Gene Ontology (GO) terms such as “Synapse Assembly” and “Axon Development” (Figure 2B; Table S1). In contrast, the hypoxia-induced module M2 (104 genes) was increased in *VCP*^*MUT*^ astrocytes in basal conditions to a similar extent as in CTRL astrocytes following hypoxia exposure, while *VCP*^*MUT*^ astrocytes exposed to hypoxic conditions showed a further increase in M2 gene expression. M2 was enriched for GO terms linked to metabolic responses, such as “Response to Oxygen Levels,” “Generation of Precursor Metabolites and Energy,” “Glucose Metabolic Process,” and “Apoptotic Mitochondrial Changes” and included genes such as *BNIP3*, *BNIP3L*, *PDK1*, *HK2*, *EGLN1*, *NOL3*, and *CAT* (Figure 2B; Table S1). M3 (36 genes) displayed no increase in *VCP*^*MUT*^ astrocytes in normoxia but an exacerbated hypoxia-associated induction in *VCP*^*MUT*^ astrocytes similar to M2. M3 was also enriched for GO terms linked to metabolic responses, including “Monosaccharide Metabolic Process” and “ADP Metabolic Process,” containing genes for glycolysis enzymes such as *PFKFB3*, *PFKFB4*, *GAPDH*, *PGK1*, *ENO1*, *ENO2*, *ALDOA*, and *HK1*. M4 (64 genes) showed a decrease in gene expression in the presence of hypoxia or the *VCP* mutation, with lowest activity in *VCP*^*MUT*^ cells exposed to hypoxia. M4 contained many mitochondrial genes, such as *MT-CO2*, *MT-CO3*, *MT-ND1*, *MT-ND2*, *MT-ATP6*, and *MT-ATP8* and was enriched for GO terms linked to mitochondrial function, including “Oxidative Phosphorylation” and “Aerobic Respiration” (Figure 2B; Table S1). Finally, M5 (65 genes), which was increased in *VCP*^*MUT*^ astrocytes while being slightly decreased by hypoxia, showed limited enrichment for genes linked to “Response to Starvation” and “Regulation of Interferon Gamma Response,” including *FAS*, *MARS1*, *PCK2*, *UCP2*, *ZP3*, *HLA-DPA1*, and *HLA-DPB1* (Figure 2B; Table S1).

Pairwise comparison of hypoxic vs. normoxic CTRL samples and normoxic *VCP*^*MUT*^ vs. CTRL samples (Figures S4A–S4C) confirmed shared upregulation of module M2 genes in both comparisons, including genes from a canonical hypoxia response signature, such as LDHA, ESPN, CA9, and AK4. To assess whether hypoxia-related gene expression changes found in our cellular model occur in astrocytes from ALS tissue, we examined the recent atlas from O’Neill et al. (2025), which identified transcriptional subclasses of ALS cases characterized by transcriptional signatures linked to oxidative/mitochondrial stress (ALS_Ox), inflammatory glial activation (ALS_Glia), and TDP-43 pathology and associated transposable elements (ALS_TE). We observed that canonical hypoxia-associated differentially expressed genes (DEGs) from our dataset (overlapping with hypoxia signatures from the Molecular Signatures Database [MSigDB] see methods) were significantly enriched among genes upregulated in astrocytes in all three ALS subclasses, most prominently the ALS_Glia subclass (Figure S5).

Overall, these analyses identified a major set of 204 hypoxia-regulated genes (M2, M3, and M4) that are substantially deregulated in *VCP*^*MUT*^ astrocytes, including genes linked to the observed alterations in mitochondrial and wider metabolic changes, as well as hypoxia-induced genes also deregulated in astrocytes in a wide spectrum of ALS cases.

### Hypoxic stress in control astrocytes is sufficient to recapitulate ALS phenotypes

**Figure 3:**
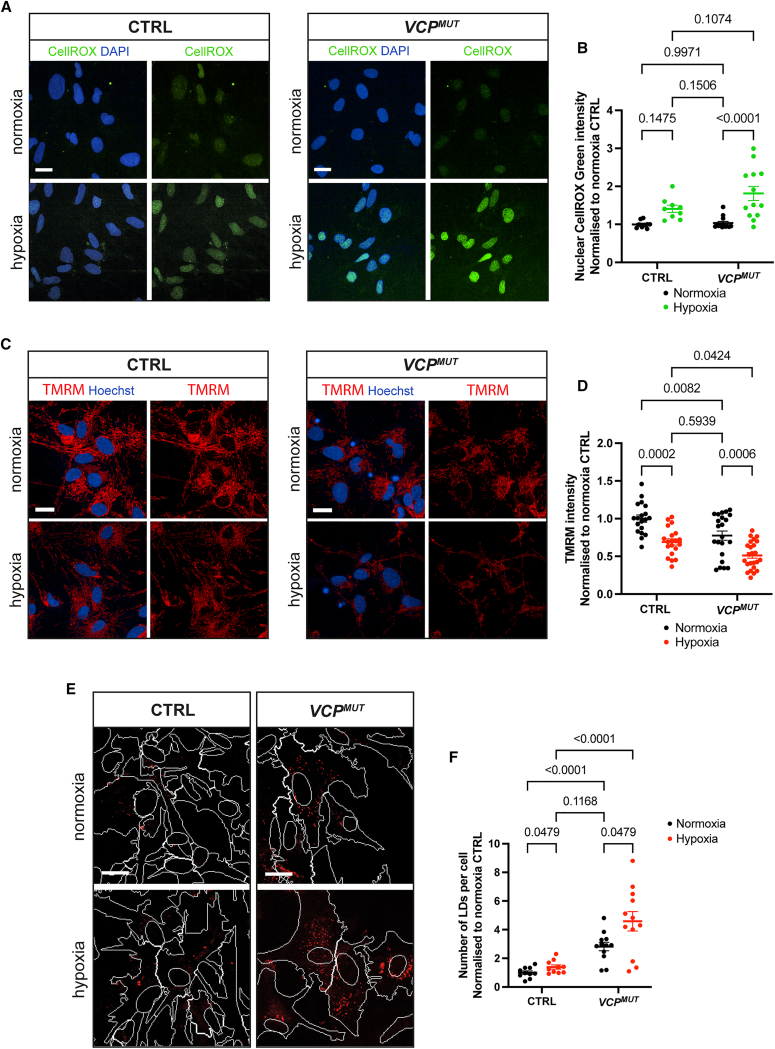
Hypoxic stress induces ALS-related phenotypes in control astrocytes (A) Representative fluorescence images of CTRL and *VCP*^*MUT*^ astrocytes under basal conditions (normoxia) and after hypoxia exposure, stained with CellROX Green (green) to visualize overall ROS generation and DAPI (blue). Scale bars, 20 μm. (B) Quantification of nuclear CellROX Green intensity, normalized to normoxia CTRL astrocytes within independent experimental repeats. Each data point represents the mean value of 20 fields across 2 technical replicates per cell line (cell lines used in Repeat 1: CTRL1, CTRL2, CTRL6, NCRM C2, NCRM E6, Mut1.1, and Mut2.1; Repeat 2: CTRL1, CTRL2, CTRL6, NCRM C2, NCRM E6, Mut2.1, and Mut2.2; Repeat 3: CTRL1, CTRL2, CTRL 6, NCRM C2, NCRM E6, Mut1.1, Mut1.2, and Mut2.1). *p* values calculated from two-way ANOVA with Tukey’s test for multiple comparisons. (C) Representative live-cell fluorescence images of CTRL and *VCP*^*MUT*^ astrocytes under normoxia and hypoxia, stained with TMRM (red) to visualize mitochondrial membrane potential, and Hoechst (blue). Scale bar, 20 μm. (D) Quantification of TMRM intensity, normalized to normoxia CTRL within independent experimental repeats. Each data point represents the mean value of 20 fields across 2 technical replicates per cell line (cell lines used in Repeat 1: CTRL2, CTRL4, CTRL6, NCRM E6, Mut1.1, Mut1.2, and Mut2.2; Repeat 2: CTRL3, CTRL5, CTRL6, Mut1.2, and Mut2.1; Repeat 3: CTRL1, CTRL2, CTRL3, CTRL6, NCRM C2, NCRM E6, Mut1.1, Mut1.2, Mut2.1, and Mut2.2). *p* value calculated from two-way ANOVA with Tukey’s test for multiple comparisons. (E) Representative fluorescence images of CTRL and *VCP*^*MUT*^ astrocytes under normoxia and hypoxia stained with Nile Red (red) to visualize LD accumulation. Nuclear (DAPI) and cytoplasmic (GFAP) masks are marked by white traces. Scale bars, 20 μm. (F) Quantification of the number of Nile Red-stained LDs per cell. Each data point represents the mean value of 10 fields per technical repeat per cell line (2 technical repeats per condition). Data normalized to CTRL normoxia within independent experimental repeats (cell lines used in Repeat 1: CTRL1, CTRL2, CTRL6, NCRM C2, NCRM E6, Mut1.1, and Mut2.1; Repeat 2: CTRL5, CTRL6, Mut1.2, and Mut2.1). *p* values calculated from two-way ANOVA with Tukey’s test for multiple comparisons. All error bars represent mean ± SEM.

Having established that *VCP*^*MUT*^ astrocytes exhibit increased HIF-1ɑ nuclear translocation, mitochondrial membrane depolarization, and LD accumulation in their basal state and deregulation at the transcriptomic level suggestive of hypoxia-associated mitochondrial and metabolic changes, we hypothesized that hypoxic stress alone is sufficient to induce these ALS-associated phenotypes in CTRL astrocytes. To test this hypothesis, we subjected CTRL astrocytes to hypoxic conditions (1% O_2_ for 24 h) using a controlled oxygen environment. We first assessed overall reactive oxygen species (ROS) generation using CellROX, which revealed no difference between CTRL and *VCP*^*MUT*^ astrocytes in their basal state (normoxia). CTRL astrocytes exposed to hypoxia showed a trend toward increased ROS production, although this increase was not statistically significant (Figures 3A, left panels, and Figure 3B). In contrast, hypoxic exposure induced a significant increase in ROS generation in *VCP*^*MUT*^ astrocytes compared to their basal state (Figures 3A, right panels, and Figure 3B). Mitochondrial health was evaluated using mitochondrial membrane potential as a proxy, as measured by TMRM staining. CTRL astrocytes subjected to hypoxia exhibited significant membrane depolarization compared to those in basal conditions (Figures 3C, left panels, and Figure 3D). Notably, hypoxic exposure exacerbated the basal mitochondrial depolarization observed in *VCP*^*MUT*^ astrocytes (Figures 3C, right panels, and Figure 3D). Given the link between mitochondrial dysfunction and lipid metabolism dyshomeostasis, we next examined LD phenotypes using Nile Red staining. Hypoxic exposure induced a significant increase in LD number in CTRL astrocytes compared to their basal state (Figures 3E, left panels, and Figure 3F). Similarly, hypoxic conditions exacerbated the LD accumulation in *VCP*^*MUT*^ astrocytes (Figures 3E, right panels, and Figure 3F). LD area, however, remained largely unaffected by hypoxic stress in both CTRL and *VCP*^*MUT*^ astrocytes (Figure S6).

### Pharmacological HIF-1ɑ stabilization in control astrocytes phenocopies ALS-associated phenotypes and reveals binding to genes involved in mitochondrial and metabolic homeostasis

**Figure 4:**
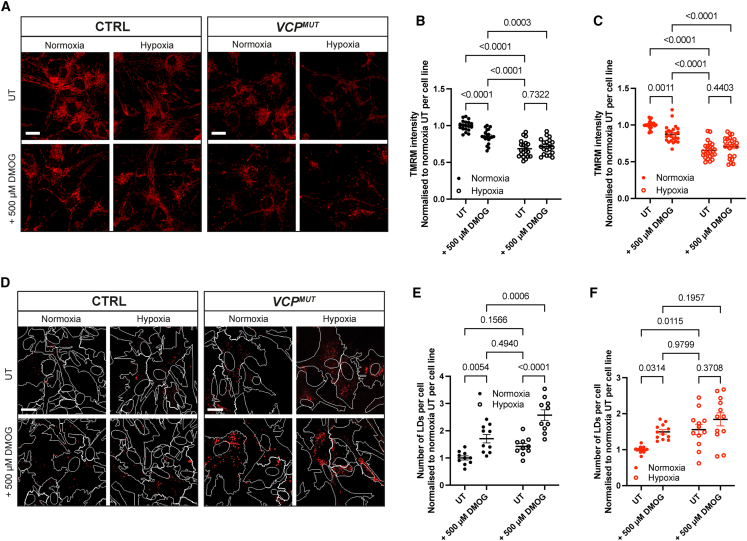
Pharmacological stabilization of HIF-1ɑ with DMOG is sufficient to phenocopy ALS-associated astrocyte phenotypes in control astrocytes (A–C) (A) Representative live-cell images of CTRL and *VCP*^*MUT*^ astrocytes under normoxia and hypoxia, with or without treatment with 500 μM DMOG, stained with TMRM (red) to visualize mitochondrial membrane potential. Scale bars, 20 μm. Quantification of TMRM intensity in (B) CTRL and (C) *VCP*^*MUT*^ astrocytes, normalized to normoxia untreated (UT) per cell line within each experimental repeat. Each data point represents the mean value of 20 fields across 2 technical replicates per cell line. Error bars represent mean ± SEM (cell lines used in Repeat 1: CTRL2, CTRL4, CTRL6, NCRM E6, Mut1.1, Mut1.2, and Mut2.2; Repeat 2: CTRL3, CTRL5, CTRL6, Mut1.2, and Mut2.1; Repeat 3: CTRL1, CTRL2, CTRL3, CTRL6, NCRM C2, NCRM E6, Mut1.1, Mut1.2, Mut2.1, and Mut2.2). *p* values calculated from two-way ANOVA with Tukey’s test for multiple comparisons. (D–F) (D) Representative fluorescence images of CTRL and *VCP*^*MUT*^ astrocytes under normoxia and hypoxia, with or without treatment with 500 μM DMOG, stained with Nile Red to visualize LD accumulation. Nuclear (DAPI) and cytoplasmic (GFAP) masks are marked by white traces. Scale bars, 20 μm. Quantification of the number of Nile Red-stained LDs per cell in (E) CTRL and (F) *VCP*^*MUT*^ astrocytes. Each data point represents the mean value of 10 fields per technical repeat (2 technical repeats per condition). Data normalized to normoxia UT per cell line within independent experimental repeats. Error bars represent mean ± SEM (cell lines used in Repeat 1: CTRL1, CTRL2, CTRL6, NCRM C2, NCRM E6, Mut1.1, and Mut2.1; Repeat 2: CTRL5, CTRL6, Mut1.2, and Mut2.1). *p* values calculated from two-way ANOVA with Tukey’s test for multiple comparisons.

To further investigate the mechanisms by which hypoxic stress induces ALS-associated phenotypes in astrocytes, we examined the role of HIF-1ɑ stabilization by employing an orthogonal method. Using DMOG, a pharmacological stabilizer of HIF-1ɑ, we found that treatment of CTRL astrocytes was sufficient to induce mitochondrial membrane depolarization (Figures 4A and 4B) and LD accumulation (Figures 4D and 4E). These findings suggest that HIF-1ɑ stabilization alone can phenocopy key ALS-related cellular abnormalities.

**Figure 5:**
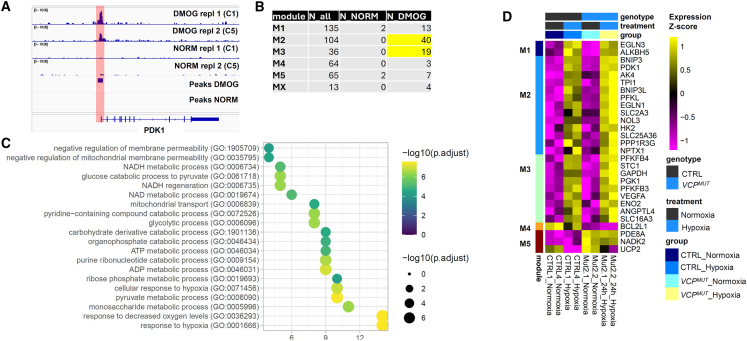
HIF-1ɑ directly binds to genes linked to alterations in mitochondrial and metabolic function in *VCP*-mutant ALS astrocytes (A) DMOG induced HIF-1ɑ binding at the PDK1 locus. (B) Overlap of HIF-1ɑ CUT&RUN peaks in DMOG-treated astrocytes with gene modules affected by 24-h hypoxia (vs. normoxia) and/or the *VCP* mutation (*VCP*^*MUT*^) vs. control (CTRL) astrocytes from Figure 2. (C) Top 20 enriched GO terms for HIF-1ɑ-bound differential genes; number of differential genes in GO term and FDR for enrichment. (D) Heatmap shows relative expression of genes that are linked to the GO terms in (C) and bound by HIF-1ɑ and affected by 24-h hypoxia (vs. normoxia) and/or the *VCP* mutation (VCPm) vs. control (CTRL) from Figure 2.

To gain deeper mechanistic insights into how the enhanced HIF-1ɑ nuclear translocation in *VCP*^*MUT*^ astrocytes under basal conditions may contribute to the observed transcriptional and cellular phenotypes of *VCP*^*MUT*^ astrocytes, we performed cleavage under targets and release using nuclease (CUT&RUN) to identify HIF-1ɑ-DNA interactions in astrocytes (Figure 5). Under basal conditions, where HIF-1ɑ nuclear localization is low (see Figure 1E), only 228 reproducible peaks of HIF-1ɑ binding to promoters were detected, while stabilizing HIF-1ɑ by DMOG treatment prominently increased DNA binding, revealing 2,425 reproducible peaks (an example being at the *PDK1* promoter, which can be seen in Figure 5A). Putative direct HIF-1ɑ target genes, defined by promoter-associated HIF-1ɑ binding, were particularly enriched among hypoxia-induced genes identified in our RNA-seq analysis (Figure 5B; see also Figure 2A). This enrichment included genes that were already upregulated in *VCP*^*MUT*^ astrocytes under basal conditions (Figure 2A, DEG module M2).

DEGs from M2, M3, and M4 (in Figure 2) that were also identified as being bound by HIF-1ɑ were enriched for GO terms such as “response to hypoxia,” “monosaccharide metabolic process,” “glycolytic process,” “mitochondrial transport,” and “negative regulation of mitochondrial membrane potential” (Figure 5C), suggesting that HIF-1ɑ binding drives the transcriptional responses associated with mitochondrial dysfunction and metabolic dysregulation observed in *VCP*^*MUT*^ astrocytes even under normoxic conditions. DEGs within M2, M3, and M4 (in Figure 2) bound by HIF-1ɑ include various glucose metabolism enzymes, e.g., *HK2*, *PFKBP3*, *PFKBP4*, *ENO2* and *PDK1*, and *BCL2* family genes controlling mitochondrial dysfunction-mediated cell death, including the pro-apoptotic genes *BNIP3* and *BNIP3L*, which are increased in *VCP*^*MUT*^ astrocytes in hypoxia, and the anti-apoptotic *BCL2* family gene *BCL2L1*, which is downregulated in *VCP*^*MUT*^ astrocytes (Figure 5D). Altogether, this indicates an altered transcriptional response to hypoxia in *VCP*^*MUT*^ astrocytes compared to CTRL astrocytes, which is linked to increased basal HIF-1ɑ activity and enhanced DNA binding. These findings suggest that aberrant HIF-1ɑ stabilization and transcriptional activity contribute to the dysregulated mitochondrial and metabolic homeostasis observed in *VCP*^*MUT*^ astrocytes.

### Hypoxic stress impairs astrocyte-mediated non-cell-autonomous RBP localization in motor neurons

**Figure 6:**
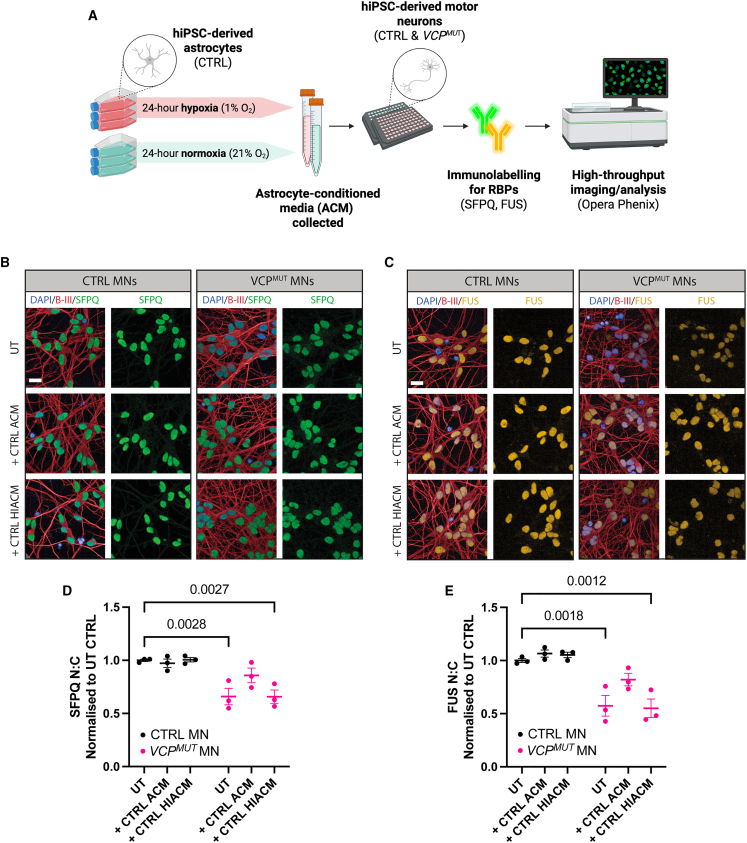
Hypoxic stress impairs the corrective effect of ACM on RBP mislocalization in *VCP*-mutant motor neurons (A) Schematic of experimental workflow. (B and C) Representative immunofluorescence images of control (CTRL) and *VCP*-mutant (*VCP*^*MUT*^) MNs under untreated (UT) conditions or following treatment with ACM derived from CTRL or hypoxic CTRL astrocytes (HIACM). MNs were stained for (B) SFPQ (green) or (C) FUS (yellow), with DAPI (blue) and βIII-tubulin (red) marking nuclei and neurites, respectively. Scale bars, 20 μm. (D and E) Quantification of N:C ratios of SFPQ (D) and FUS (E), normalized to untreated CTRL MNs. Data represent mean ± SEM across three independent CTRL and three independent *VCP*^*MUT*^ astrocyte-MN line pairs, from one independent experimental repeat (cell lines used: CTRL3, CTRL4, CTRL5, NCRM C2, Mut2.1, and Mut2.2). *p* values calculated from two-way ANOVA with (D) Šídák’s test for multiple comparisons and (E) Dunnett’s test for multiple comparisons.

We finally asked whether hypoxic stress alters the ability of astrocytes to regulate nuclear to cytoplasmic (N:C) RBP localization in MNs. To do this, we exposed CTRL and *VCP*^*MUT*^ MNs to astrocyte-conditioned medium (ACM) from untreated/normoxic CTRL astrocytes and those exposed to 24-h hypoxia (Figure 6A). *VCP*^*MUT*^ MNs displayed significant N:C mislocalization of SFPQ and FUS under basal conditions (Figures 6B and 6C, top right panels; Figures 6D and 6E). ACM from CTRL astrocytes exerted a corrective effect, significantly restoring N:C partitioning of both RBPs in *VCP*^*MUT*^ MNs (Figures 6D and 6E). These findings are consistent with those of our previous study demonstrating a similar corrective capacity of astrocytes in the context of TDP-43 proteinopathy in MNs (Smethurst et al., 2020). By contrast, ACM from hypoxia-exposed CTRL astrocytes failed to confer this correction, indicating that hypoxic stress disrupts the supportive capacity of astrocytes to non-cell-autonomously regulate RBP localization in MNs.

## Discussion

In this study, we demonstrate that human ALS astrocytes carrying *VCP* mutations exhibit early, cell-autonomous activation of the hypoxia response pathway. This is evidenced by the increased nuclear translocation of HIF-1ɑ under basal conditions, even in normoxia, and HIF-1ɑ-dependent mitochondrial membrane depolarization and LD accumulation. Through RNA-seq and CUT&RUN profiling, we confirm that this aberrant HIF-1ɑ activity drives the transcription of canonical hypoxia target genes, particularly those linked to mitochondrial and metabolic stress. These include glycolytic enzymes, redox regulators, and pro-apoptotic mediators. Notably, many of these targets were bound directly by HIF-1ɑ in *control* astrocytes under hypoxic conditions, as revealed by CUT&RUN, and their expression in *VCP* mutants mimicked that of hypoxia-exposed control astrocytes. This suggests that *VCP*-mutant astrocytes exist in a pseudo-hypoxic state. Noting this elevated basal activity, *VCP*-mutant astrocytes displayed a somewhat attenuated transcriptional response to further hypoxic challenge. Importantly, we show that this aberrant hypoxia response is not epiphenomenal, but functionally relevant: exposing control astrocytes to hypoxia—or pharmacologically stabilizing HIF-1ɑ using DMOG—was sufficient to phenocopy *VCP*-mutant phenotypes, including mitochondrial membrane depolarization and LD accumulation. These findings implicate persistent HIF-1ɑ activation as a key upstream driver of metabolic dysfunction in ALS astrocytes.

Nomura et al. (2019) reported that repeated intraperitoneal DMOG administration was protective in SOD1^G93A^ mice, likely reflecting systemic, multi-cellular adaptations and the well-established phenomenon of preconditioning that arises from repeated exposure to hypoxic stimuli. By contrast, in our study, DMOG was used to directly assess the consequences of HIF-1ɑ stabilization in human iPSC-derived astrocytes. The different outcomes therefore likely reflect distinctions in species (mouse vs. human), experimental context (systemic vs. cell instrinsic), treatment paradigm (repeated preconditioning vs. single direct stabilization), and genetic model (*SOD1* vs. *VCP*).

### Astrocytic hypoxic stress in ALS

While systemic hypoxia resulting from respiratory muscle degeneration is a well-documented terminal feature of ALS, our findings suggest that astrocytes experience intrinsic hypoxic stress much earlier in disease. This occurs independently of neuronal degeneration or any change in systemic oxygen levels and likely reflects metabolic vulnerabilities or dysregulated oxygen sensing induced by ALS-causing mutations. Possible triggers include increased mitochondrial oxygen demand, impaired oxygen diffusion, dysfunctional redox buffering, or altered regulation of HIF degradation pathways—each of which present important avenues for further investigation.

Our results align with, and extend, previous studies reporting elevated HIF-1ɑ in ALS spinal cord tissue, reduced VEGF expression, and cell-type-specific differences in hypoxia responses (Nagara et al., 2013; Wiesner et al., 2013). Notably, in a SOD1 mouse model from the same study, impaired transport of HIF-1ɑ from the cytoplasm to the nucleus was observed in MNs, suggesting dysregulation at the level of nuclear translocation as a potential mechanism for altered HIF-1ɑ function. In other SOD1 models, astrocytes display transient activation of hypoxic programs at early presymptomatic stages, followed by diminished responsiveness with disease progression (Nomura et al., 2019), consistent with our observation of blunted HIF-1ɑ reactivity in *VCP*-mutant astrocytes. Furthermore, our recent meta-analysis identified upregulation of hypoxia-related pathways as a convergent feature across mouse and human ALS transcriptomic datasets (independent of disease-causing mutation) (Ziff et al., 2022). The enrichment of our hypoxia-responsive genes across O’Neill et al.’s ALS_Ox, ALS_Glia, and ALS_TE modules reinforces that hypoxia pathway activation intersects with oxidative/mitochondrial stress, inflammatory reactivity, and TDP-43-linked pathology. Together with Ziff et al. (2022), these findings highlight hypoxia signaling as a conserved and recurrent hallmark of ALS astrocytes across genetic and pathological contexts.

### HIF-1ɑ links hypoxia to mitochondrial dysfunction

Mitochondria are both central consumers of oxygen and regulators of redox state, making them particularly susceptible to hypoxia. In astrocytes, hypoxia has been shown to impair respiratory chain activity and reduce tricarboxylic acid (TCA) cycle flux (Allen et al., 2020). We show that *VCP*-mutant astrocytes exhibit significant mitochondrial membrane depolarization under normoxic conditions—a phenotype recapitulated in control astrocytes exposed to hypoxia or to the HIF-1ɑ stabilizer DMOG. These data implicate chronic, cell-autonomous HIF-1ɑ activation in mitochondrial dysfunction in ALS astrocytes. Mechanistically, HIF-1ɑ has been shown to modulate mitochondrial biology through multiple pathways: promoting a metabolic shift from oxidative phosphorylation to glycolysis, reducing mitochondrial biogenesis and respiration, and upregulating PDK1, which inhibits pyruvate entry into the TCA cycle (Huang et al. 2022). Consistent with this, our RNA-seq analysis revealed that *PDK1*, along with other key metabolic regulators including *BNIP3*, *BNIP3L*, *HK2*, *EGLN1*, and *CAT*, were upregulated in *VCP*-mutant astrocytes under basal conditions—mirroring their expression in hypoxia-exposed controls. Notably, many of these genes were also bound by HIF-1ɑ in our CUT&RUN dataset, suggesting direct transcriptional regulation. These genes clustered primarily within RNA-seq modules 2 and 3, which were enriched for GO terms related to “mitochondrial membrane potential,” “apoptotic mitochondrial change,” and “generation of precursor metabolites and energy.” Their expression was already elevated in *VCP*-mutant astrocytes at baseline and further amplified under hypoxia, suggesting a hyperactive and potentially maladaptive hypoxic transcriptional program. In parallel, module 4—encompassing numerous mitochondrial-encoded genes (*MT-CO2*, *MT-CO3*, *MT-ATP6*, *MT-ND1*)—showed decreased expression in both *VCP*-mutant and hypoxia-exposed astrocytes, indicative of mitochondrial respiratory compromise.

Furthermore, *VCP*-mutant astrocytes displayed increased susceptibility to ROS accumulation under hypoxia, despite no differences at baseline. This heightened vulnerability may reflect weakened antioxidant defenses, possibly involving dysregulation of the KEAP1-NRF2 pathway—a major redox regulatory system known to be impaired in ALS (Dinkova-Kostova et al. 2018). While our use of CellROX allowed for global ROS detection, future studies with mitochondrial-targeted ROS probes (e.g., MitoSOX) will be important to determine whether the observed oxidative stress arises directly from dysfunctional mitochondria. Altogether, these findings provide converging evidence—across live-cell imaging, transcriptional profiling, and HIF-1ɑ chromatin binding—that mitochondrial dysfunction in ALS astrocytes is tightly coupled to chronic HIF-1ɑ activation. They also suggest that aberrant activation of the hypoxia response contributes to a feedforward loop of metabolic disruption and oxidative stress, compounding astrocyte vulnerability and likely impairing their neuroprotective roles.

### HIF-1ɑ drives lipid dyshomeostasis via LD accumulation

Another key feature of ALS astrocytes in our model is the accumulation of LDs, which was observed at baseline in *VCP*-mutant cells and was further exacerbated by hypoxia or DMOG. While hypoxia-induced LD accumulation is a known adaptive response to store fatty acids for β-oxidation during glucose scarcity (Welte 2015), the chronicity and magnitude of accumulation in *VCP*-mutant astrocytes—in addition to the increased LD size—suggests a maladaptive shift in lipid homeostasis. VCP plays a role in endoplasmic reticulum- (ER)-associated degradation and autophagy (Chou and Deshaies 2011; Xia et al. 2016), which intersect with lipid metabolism. Disruption of these pathways may promote excess lipid storage, impaired lipophagy, or altered LD maturation. Additionally, the larger LD size in *VCP*-mutant astrocytes suggests defects in LD fusion, composition, or breakdown, which could alter their functional roles in stress signaling and inflammation (Olzmann and Carvalho 2019). Notably, while our CUT&RUN data primarily identified HIF-1ɑ binding at genes regulating glycolysis, mitochondrial function, and oxidative stress—such as *PDK1*, *HK2*, *BNIP3*, and *BNIP3L*—the phenotypic induction of LD accumulation by both hypoxia and DMOG supports a functional role for HIF-1ɑ in lipid metabolic reprogramming. Whether this regulation occurs through direct transcriptional control of lipid homeostasis genes remains an important question for future work.

The functional consequences of LD accumulation are not benign. LD-rich astrocytes display impaired mitochondrial respiration and can release neurotoxic factors (Windham et al., 2023; Kwon et al., 2017; Zhang et al., 2022; Liu et al., 2015; Lee et al., 2017). In other models, pharmacological reduction of LDs via CB2R activation restores mitochondrial function and reduces inflammation (Cruz et al., 2020). The ability of DMOG to induce LD accumulation even in the absence of hypoxia confirms that HIF-1ɑ activation is sufficient to drive lipid dyshomeostasis, further hinting toward its central role in the pathophysiology of ALS astrocytes. Together, these findings suggest a feedforward loop, whereby HIF-1ɑ-mediated LD accumulation impairs mitochondria, further exacerbating oxidative stress and reinforcing the pathological state.

### An integrated hypoxia-metabolism axis in ALS astrocytes

Our multi-modal approach—combining phenotypic assays, transcriptomics, and protein-DNA interaction profiling—reveals a convergent axis of hypoxia, mitochondrial dysfunction, and lipid dyshomeostasis in *VCP*-mutant astrocytes. Our findings are consistent with recent *in vivo* evidence that astrocytic mitochondrial dysfunction impairs fatty acid degradation and drives LD accumulation and neurodegeneration (Mi et al., 2023), reinforcing the coupling of mitochondrial- and lipid-mediated stress as a core pathogenic axis.

Moreover, by identifying a set of HIF-1ɑ-bound and hypoxia-responsive genes—including *BNIP3*, *HK2*, *PFKFB3/4*, and *PDK1*—as dysregulated in ALS astrocytes, our data point toward new targets for therapeutic modulation of the hypoxia response. Whether interventions aimed at rebalancing HIF-1ɑ activity can restore metabolic function and delay neurodegeneration remains a critical question for future studies. While our study focused on *VCP-*mutant astrocytes, the implications may extend more broadly across ALS. Several ALS-associated genes, including *SOD1*, *FUS*, and *C9orf72*, are known to play canonical roles in oxygen sensing, regulating mitochondrial integrity, oxidative stress responses, and metabolic homeostasis (Reddi and Culotta 2013; Tsai et al., 2020; Wang et al., 2021). This convergence suggests that impaired adaptation to hypoxic or bioenergetic stress could represent a shared pathogenic mechanism across diverse ALS genotypes. Our findings provide a mechanistic framework to explore whether dysregulated HIF-1ɑ signaling and hypoxia pathway activation also contribute to astrocyte dysfunction in other familial or sporadic contexts.

### Hypoxic stress compromises astrocytic regulation of motor neuron RBP localization

We have shown that *VCP*-mutant astrocytes experience intrinsic hypoxic stress, characterized by HIF-1ɑ stabilization, mitochondrial dysfunction, and lipid dyshomeostasis. Our RNA-seq and CUT&RUN analyses further revealed that this stress is underpinned by transcriptional reprogramming, with HIF-1ɑ directly regulating genes linked to metabolic disruption and oxidative stress. However, astrocytes do not act in isolation within the CNS—their relevance to ALS lies in their ability to support or impair MN health. We, therefore, asked how hypoxic stress in astrocytes influences a hallmark neuronal phenotype in ALS—the mislocalization of RBPs.

Our ACM experiments demonstrate that healthy astrocytes secrete factors that can significantly correct the mislocalization of SFPQ and FUS in *VCP*-mutant MNs, consistent with a supportive role of non-diseased astrocytes. Strikingly, this corrective capacity was lost when astrocytes were exposed to hypoxia, indicating that hypoxic stress reprograms the astrocytic secretome in a manner that reduces their ability to support MN homeostasis. This provides functional evidence that astrocytic hypoxic stress not only drives intrinsic dysfunction but also directly compromises their non-cell-autonomous influence on motor neurons. Together with our astrocyte-intrinsic findings, these data underscore that hypoxia-induced alterations in astrocytes have impact beyond cell-autonomous changes, impairing astrocyte-neuron interactions that are central to disease progression.

### Conclusion

Our study sheds new light on the role of hypoxic signaling in ALS by uncovering a previously unrecognized, cell-intrinsic activation of the hypoxia pathway in human astrocytes harboring *VCP* mutations. We provide the first direct evidence that HIF-1ɑ accumulates in the nucleus under basal conditions in ALS astrocytes, accompanied by a transcriptional program indicative of metabolic stress and mitochondrial dysfunction. This reveals that glial HIF-1ɑ dysregulation is not a merely a downstream consequence of disease progression or systemic hypoxia, but an early, pathogenic event that may actively drive astrocyte dysfunction in ALS.

Through phenotypic assays, transcriptomic profiling, and CUT&RUN analysis, we demonstrate that chronic activation of the hypoxia pathway in *VCP*-mutant astrocytes contributes to key hallmarks of astrocyte pathology in ALS—mitochondrial depolarization, LD accumulation, and likely metabolic reprogramming. Extending these findings to a neuron-glial communication context, we further show that hypoxic stress impairs the ability of astrocytes to support MNs, linking intrinsic astrocytic dysfunction to a canonical neuronal hallmark of ALS (RBP mislocalisation). Together, these findings position HIF-1ɑ as a central upstream regulator of astrocyte pathology and highlight hypoxia-induced metabolic stress as a possible driver of disrupted neuron-glia interactions in ALS. Future studies should explore whether targeted modulation of HIF-1ɑ signaling can restore astrocyte homeostasis and protect against neurodegeneration.

## Methods

### Ethics statement

Informed consent was obtained from all patients and healthy controls who donated samples for hiPSC culture. Experimental protocols were conducted according to approved regulations and guidelines by University College London (UCL) Hospitals’ National Hospital for Neurology and Neurosurgery and UCL’s Institute of Neurology joint research ethics committee (09/0272).

### Derivation of human fibroblasts and hiPSC generation

hiPSC lines included 7 control lines (CTRL1–6 and one where we corrected the R155C mutant line to R155R) and 4 ALS *VCP*-mutant lines, including 2 clones of R155C from one patient and 2 clones of R191Q from another patient. Furthermore, we included 2 additional isogenic *VCP*-mutant lines generated through knockin of the R191Q mutation. Three of the control lines are commercially available and were purchased from Coriell (ND41866^∗^C), ThermoFisher Scientific (A18945), and Cedars Sinai (CS02iCTR-NTn4). CTRL1 and the ALS *VCP*-mutant lines were kindly donated by Professor Selina Wray and her lab. They collected patient dermal fibroblasts and cultured them in OptiMEM +10% fetal calf serum medium. For hiPSC generation, transfection of the following episomal plasmids was performed: pCXLE hOct4 shp53, pCXLE hSK, and pCXLE hUL (Addgene) (Okita et al., 2011). Details for all hiPSC lines utilized in this paper can be found in Table S2.

### hiPSC maintenance

All hiPSC lines were maintained in feeder-free, chemically defined monolayers on Geltrex (ThermoFisher) basement membrane matrix in Essential 8 Medium (ThermoFisher) under standard incubation conditions (37°C, 5% CO_2_, and 21% O_2_). Cells were passaged at ∼70% confluency. Further information on routine maintenance, passaging, cryopreservation, and thawing is provided in Method S1.

### hiPSC-derived neural precursor generation

For differentiation to neuroepithelium, E8 media was switched to neural induction media containing a 1:1 ratio of maintenance media, N2 and B27, and supplemented with dorsomorphin, SB431542, and CHIR99021 for 7 days. At day 5, the neuroepithelial layer was enzymatically dissociated using dispase and replated. To caudalize and ventralize cells to the motor neuron progenitor (pMN) domain of the spinal cord, neural induction media was replaced with patterning media, consisting of maintenance media supplemented with retinoic acid and purmorphamine for a further 7 days before a 4-day phase in maintenance media and reduced purmorphamine only. During this phase, cells were expanded using dispase if necessary. After patterning and prior to terminal differentiation, neural precursor cells (NPCs) were expanded to increase cell material by using maintenance media supplemented with fibroblast growth factor (FGF)-2 for up to 30 days, cryopreserved in DMSO for use in future experiments, or subjected to terminal differentiation. During this stage, cells were split using EDTA and plated onto Geltrex-coated plates. Full media composition, reagent concentrations, incubation times, and plating densities are described in Method S1.

### Astrocyte differentiation from hiPSC-derived NPCs

For differentiation to astrocytes, NPCs were propagated further in maintenance media with 10 ng/mL FGF-2 (Peprotech) for 60–120 days to generate glial precursor cells (GPCs). Cells were split using Accutase and maintained on Geltrex-coated 6-well plates or T25/T75 flasks. Terminal differentiation was achieved with maintenance media and 10 ng/mL bone morphogenetic protein 4 (BMP4) (R&D) and 10 ng/mL leukemia inhibitory factor (LIF) (Sigma-Aldrich) for 21 days followed by 7 days in maintenance media only. The latter is an adaptation to our original protocol (Hall et al., 2017), where differentiation for 28 days was undertaken in BMP4 and LIF. In this revised version, astrocytes are instead given 7 days without BMP4 and LIF, allowing them time to resume a more unstimulated state prior to experimentation. For final plating, cells were dissociated with Accutase and counted before plating in maintenance media into required formats on Geltrex-coated plates. Typical plating densities are provided in Method S1.

### Motor neuron differentiation and plating

Directed differentiation into MNs was carried out as per protocol outlined by Hall et al., (2017). For differentiation to neuroepithelium, E8 media was switched to neural induction media containing a 1:1 ratio of maintenance media, N2 and B27, and supplemented with dorsomorphin, SB431542, and CHIR99021 for 7 days. At day 5, the neuroepithelial layer was enzymatically dissociated using dispase and replated. To caudalize and ventralize cells to the pMN domain of the spinal cord, neural induction media was replaced with patterning media, consisting of maintenance media supplemented with 0.5 μM retinoic acid (Sigma-Aldrich) and 1 μM purmorphamine (Merck Millipore) for a further 7 days before a 4-day phase in maintenance media and reduced purmorphamine (0.1 μM) only. During this phase, cells were expanded using 1 mg/mL dispase if necessary. After patterning and prior to terminal differentiation, NPCs were either expanded to increase cell material by using maintenance media supplemented with 10 ng/μL FGF-2 (Peprotech) for up to 30 days, snap-frozen for use in future experiments, or subjected to terminal differentiation. During this stage, cells were split using EDTA and plated onto Geltrex-coated plates. For final plating, NPCs were dissociated with Accutase (ThermoFisher) and plated into different formats on polyethylenimine and Geltrex-coated plates. NPCs were counted and plated in maintenance media with 10 ng/μL FGF-2 and supplemented with 10 μM of ROCK inhibitor (Y-27632). The following day, NPCs were terminally differentiated in maintenance media and 0.1 μM Compound E (Enzo Life Sciences) to promote cell cycle exit and generate synchronized, terminally differentiated, and post-mitotic MNs. Plating conditions and coating procedures are described in Method S1.

### Hypoxia treatment

Cells were either maintained at 37°C, 5% CO_2_, and 21% O_2_ (normoxia), or at 1% O_2_ (hypoxia). The hypoxic environment was created by use of an SCI-tive hypoxia workstation (Ruskinn Technology). All cells underwent a fresh media change prior to hypoxia treatment, and all experimental manipulations were performed inside the workstation to avoid capturing effects of reoxygenation. When possible, cells were also fixed or harvested while still within the hypoxic environment. All cells were cultured for the same period of time, with plates being transferred from a humidified incubator, thus normoxia (21% O_2_), to the hypoxia chamber (1% O_2_) for the desired duration with all plates (including those kept in normoxia) being collected at the same time endpoint. Hypoxia mimetic DMOG (Sigma-Aldrich) was reconstituted in H_2_O at a concentration of 30 mg/mL and added to maintenance media immediately prior to treatment with a final concentration of 500 μM.

### ACM preparation and motor neuron treatment

Spent ACM was collected per cell line after 24-h incubation under either normoxia or hypoxia and immediately snap-frozen before storage at −80°C. Before use, ACM samples were thawed at room temperature (RT) and centrifuged to remove cell debris. Equal volumes of clean supernatant from individual CTRL lines were pooled together per condition to make CTRL ACM and CTRL HIACM (hypoxia-induced astrocyte conditioned media), respectively. ACM was added to hiPSC-derived MNs on day 7 of the established differentiation protocol, unless otherwise specified, in a 70:30 ratio with fresh maintenance media supplemented with Compound E (1:10,000). Further handling details are described in Method S1.

### High-throughput cellular health and imaging assays

Astrocytes were assessed for mitochondrial membrane potential, mitochondrial area, LD accumulation, intracellular ROS, and immunocytochemical markers using live or fixed high-throughput imaging. Cells were plated in 96-well formats and maintained under normoxic or hypoxic conditions in the absence or presence of drug treatments, stained with the appropriate fluorescent probes, and visualized using the PerkinElmer Opera Phenix High Content Screening System. For each well, a minimum of 8 fields were acquired. Images were analyzed with the complementary Columbus Image Data Storage and Analysis system. Detailed staining protocols, dye preparations, acquisition parameters, segmentation strategy, and antibody information are provided in Method S1 and Table S3.

### RNA sequencing sample preparation

Poly(A)+-selected reverse-stranded RNA-seq libraries were prepared from 2 control and 2 *VCP*-mutant lines, under basal conditions (normoxia) or after exposure to 24-h 1% O_2_ hypoxia, using the KAPA mRNA HyperPrep Library kit for Illumina, with 50 ng of total RNA as input. Libraries were sequenced on the NovaSeq 6000 platform.

### CUT&RUN sample preparation

hiPSC-derived astrocytes were left untreated or treated with 500 μM DMOG for 24 h, lightly fixed, and processed using the Cell Signaling CUT&RUN assay kit (#86652) according to the manufacturer’s instructions, followed by Illumina-compatible library preparation and NovaSeq 6000 sequencing at 8 million 100-bp paired end reads per sample. Full experimental details including fixation, bead binding, MNase digestion, adapter ligation, amplification, and clean-up steps are provided in Method S1.

### Computational analysis of RNA-seq and CUT&RUN

RNA-seq reads from fastq files were mapped to the human genome (GRCh38) using the nf-core/rnaseq nextflow pipeline (v.3.5, doi: https://doi.org/10.5281/zenodo.1400710). After removing lowly expressed genes (≤ 0.5 counts per million), differential expression analysis was performed with DESeq2 (v.1.46.0), using a model accounting for genotype and treatment with the commands DESeqDataSetFromMatrix(… design = ∼treatment^∗^genotype) and DESeq(dds, test = “LRT”, reduced = ∼1), or, for pairwise comparisons (Figure S4), using DESeqDataSetFromMatrix(…, design = ∼group). DEGs (FDR ≤ 0.05) were then grouped into co-expressed modules using the function degPatterns(minc = 10, time = “treatment”, col = “genotype”) from the Bioconductor R package DEGreport (v1.42.0, DOI: https://doi.org/10.18129/B9.bioc.DEGreport) on the vst-normalized expression matrix. Functional enrichment analyses for GO terms and gene sets from the MSigDB was performed using the Bioconductor R packages clusterProfiler (v.4.14.1) with org.Hs.eg.db (v3.20.0), DOSE (v.4.0.0), and msigdbr (v7.5.1). As a broad signature of canonical hypoxia-regulated genes, we used genes occurring in any of the following hypoxia gene sets from the MSigDB—“HALLMARK_HYPOXIA,” “GOBP_RESPONSE_TO_OXYGEN_LEVELS,” “QI_HYPOXIA”, “HARRIS_HYPOXIA,” “LEONARD_HYPOXIA,” “KIM_HYPOXIA.”

CUT&RUN fastq files were analyzed using the nf-core/cutandrun pipeline (v.3.2.2, doi: https://doi.org/10.5281/zenodo.10606804). Reads were mapped to the human genome GRCh38 (hg38) using Bowtie2 (Langmead and Salzberg 2012). We used Picard (McKenna et al., 2010) to mark duplicate reads, and SAMtools (Li et al., 2009) was used to convert and index SAM files into BAM files. Reads were also aligned to the *E. coli* K12-MG1655 reference genome and spike-in normalization was performed using BEDtools (Quinlan and Hall 2010). SEACR (Meers et al. 2019) was used to call peaks. Peaks were annotated using the R Package ChiPseeker (Ramírez et al., 2016) with the transcript database TxDb.Hsapiens.UCSC.hg38.knownGene as the input.

Full parameter settings, spike-in normalization, H3K4me3 overlap, and peak filtering strategy are described in Method S1.

## Resource availability

### Lead contact

Requests for further information and resources should be directed to and will be fulfilled by the lead contact, Rickie Patani (rickie.patani@nus.edu.sg).

### Materials availability

This study did not generate unique reagents.

### Data and code availability

• All data reported in this paper will be shared by the lead contact upon request.
• This paper does not report original code.
• Any additional information required to reanalyze the data reported in this paper is available from the lead contact upon request.

## Acknowledgments

We thank Selina Wray and her laboratory for the generous donation of patient-derived fibroblasts and iPSC lines, and we are especially grateful to the individuals who kindly donated skin biopsies for the derivation of ALS and control hiPSC lines used in this study. We thank all members of the Patani lab for their valuable feedback and technical support. We gratefully acknowledge the Francis Crick Institute’s High Throughput Screening Science Technology Platform for imaging support, and the Advanced Sequencing Facility for the preparation of RNA-seq and CUT&RUN libraries. During the period in which this study was conducted, H.D.F. was supported by a 10.13039/501100000265Medical Research Council PhD studentship (MRC DTP: MR/N013867/1). R.P. gratefully acknowledges generous support from a Lister Research Prize Fellowship, Steve Redgwell, Liane Iles, Challenging MND, the Motor Neuron Disease Association (Patani/Dec22/957-793), My Name’5 Doddie Foundation (MN5DF/2022/003), and 10.13039/100013013Target ALS (BB-2024-C4-L4). This work was also supported by the 10.13039/100010438Francis Crick Institute, which receives its core funding from 10.13039/501100000289Cancer Research UK, the 10.13039/501100000265UK Medical Research Council, and the 10.13039/100010269Wellcome Trust.

## Author contributions

H.D.F. and R.P. conceived the project. R.P. supervised the study and provided strategic guidance throughout the PhD during which this work was undertaken. H.D.F designed and performed the majority of experiments, including cell culture, astrocyte and motor neuron differentiation, hypoxia treatments, phenotypic assays, imaging, data analysis, and visualization. B.E.C. and S.M. contributed to cell culture and astrocyte differentiation. H.C. led the preparation of CUT&RUN samples, including extensive troubleshooting, while N.P. and H.P. carried out initial bioinformatic analyses of the CUT&RUN dataset. M.L. performed the RNA-seq analysis and integrated the transcriptomic and CUT&RUN datasets. M.H. provided technical expertise on high-throughput imaging using the Opera Phenix system and supported image processing and analysis. S.J.B. offered critical input on chromatin biology. H.D.F. wrote the manuscript with input and revisions from all authors. All authors read and approved the final manuscript.

## Declaration of interests

The authors declare no competing interests.

## Supplemental information

Document S1. Figures S1–S6, Tables S2–S4, and Methods S1

Table S1. RNA-seq module gene list, related to Figure 2

Document S2. Article plus supplemental information
