# Supplementary material for: Hypoxic stress is an early pathogenic event in human VCP-mutant ALS astrocytes
Source: Stem Cell Reports. 2025 Dec 4;21(1):102723. doi: 10.1016/j.stemcr.2025.102723 (PMC12925969; doi:10.1016/j.stemcr.2025.102723)
Supplement: Document S1. Figures S1–S6, Tables S2–S4, and Methods S1 [file mmc1.pdf]

Stem Cell Reports, Volume *21*

Supplemental Information

Hypoxic stress is an early pathogenic event in human *VCP*-mutant ALS astrocytes

Hannah D. Franklin, Hamish Crerar, Nishita Parnandi, Michael Lattke, Stanislaw Majewski, Benjamin E. Clarke, Husayn Pallikonda, Michael Howell, Simon J. Boulton, and Rickie Patani

Supplementary Material

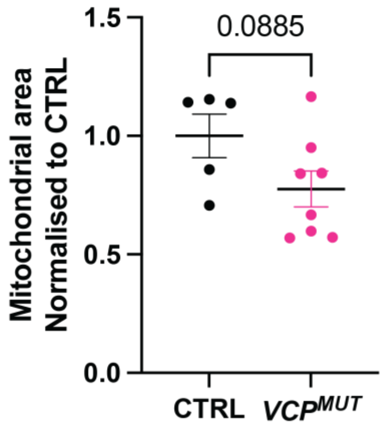

Supplementary Figure S1. HiPSC-derived VCP-mutant ALS astrocytes display a non-significant trend towards reduced mitochondrial area. Quantification of mitochondrial area in CTRL and *VCP*^{MUT} astrocytes stained with MitoTracker Green, normalised to CTRL within experimental repeat. Data shown from two independent experimental repeats (cell lines used in Repeat 1: CTRL1, CTRL4, CTRL5, NCRM C2, NCRM E6, Mut1.1, Mut1.2, Mut2.2; Repeat 2: CTRL1, CTRL5, NCRM E6, Mut1.1, Mut2.2). Each data point represents the mean value per cell line per experimental repeat (30 fields across three technical replicates). P-value calculated from unpaired t-test.

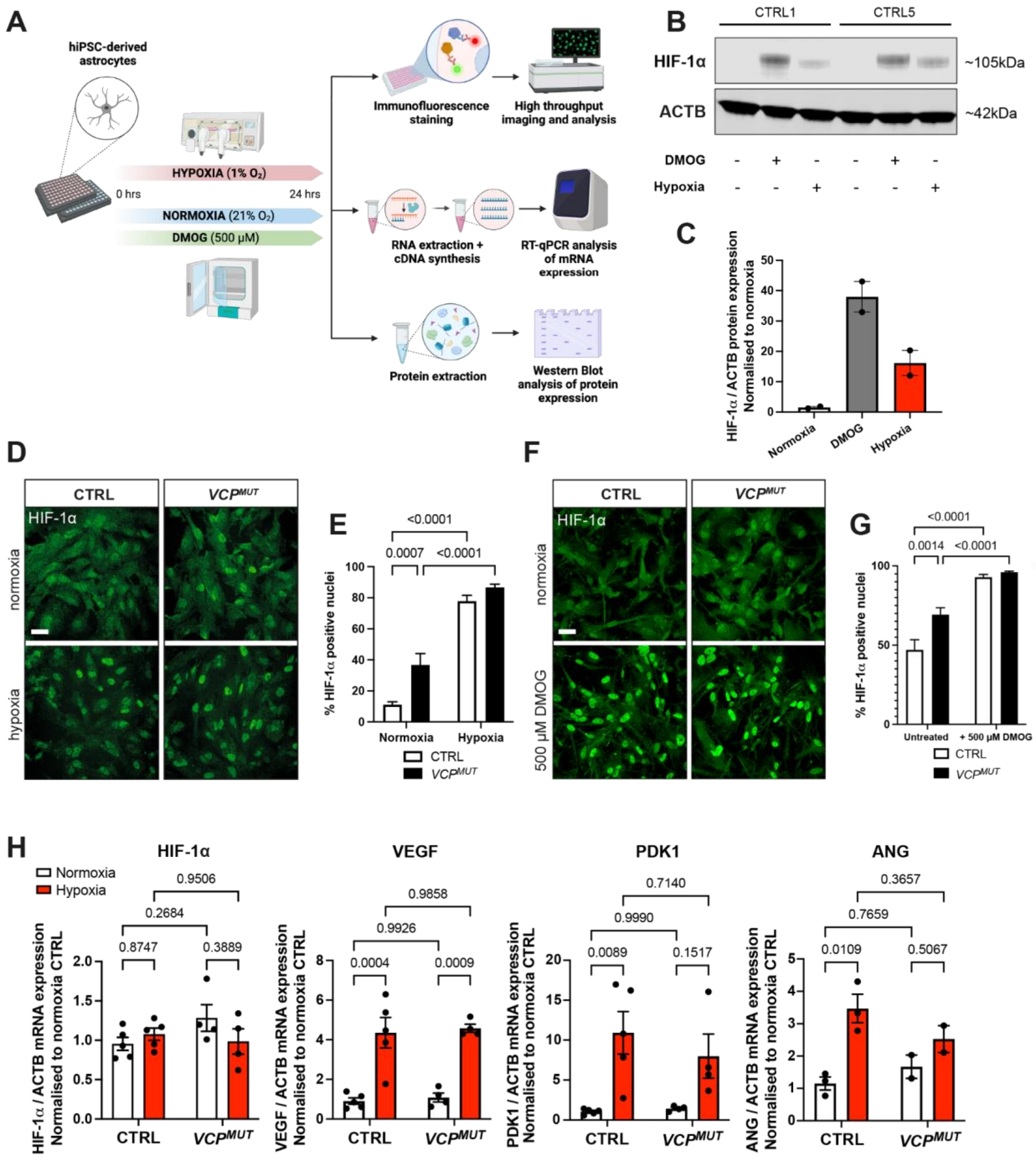

Supplementary Figure S2. Validation of hypoxia pathway activation in hiPSC-derived control and VCP-mutant astrocytes treated with DMOG or exposed to 1% O_2. **(A)** Schematic illustration of steps taken to validate an in vitro paradigm to induce hypoxia activation in hiPSC-derived astrocytes. **(B)** Western blot for whole-cell HIF-1α protein expression in two CTRL hiPSC-derived astrocyte lines maintained under normoxic conditions, treated with 500 µM DMOG or 24-

hour hypoxia (1% O_2). **(C)** Quantification of relative protein HIF-1α expression from western blot analysis, normalised to ACTB. Data expressed as fold change from normoxia. **(D-G)** Representative fluorescence images of CTRL and *VCP*^{MUT} hiPSC-derived astrocytes maintained under normoxia and exposed to **(D)** 24-hour hypoxia (1% O_2) or **(F)** 24-hour treatment with 500 μM DMOG, immunolabelled with HIF-1α (green). Scale bars: 40 μm. Bar plots depicting quantitative immunofluorescence cell-by-cell analysis of the % of nuclei exhibiting cytoplasmic-to-nuclear translocation of HIF-1α in response to **(E)** 24-hour hypoxia (1% O_2) and **(G)** 24-hour treatment with 500 μM DMOG. Data are representative of 10 fields acquired per technical replicate, 2 technical repeats per cell line (cell lines used in Repeat 1: CTRL1, CTRL4, VCPF10, Mut 2.1, Repeat 2: CTRL1, CTRL4, Mut 1.1, Mut2.1, Repeat 3: CTRL4, VCPF10, Mut2.2). *P*-values calculated from two-way ANOVA with Tukey's test for multiple comparisons. **(H)** Bar plots depicting qPCR analysis for whole-cell expression of HIF-1α and downstream target genes VEGF, PDK1 and ANG, normalised to expression of housekeeping gene ACTB, in CTRL and *VCP*^{MUT} hiPSC-derived astrocytes exposed to 24-hour hypoxia (1% O_2). Data are presented as mean value of 3 technical repeats per cell line ± SEM, normalised to normoxia CTRL within each repeat (cell lines used in Repeat 1: CTRL1, CTRL4, Mut2.1, Mut2.2, Repeat 2: CTRL5, CTRL6, Mut1.2, Mut2.1). *P*-values calculated from two-way ANOVA with Tukey's test for multiple comparisons. For all graphs, each data point represents the mean value per cell line ± SEM.

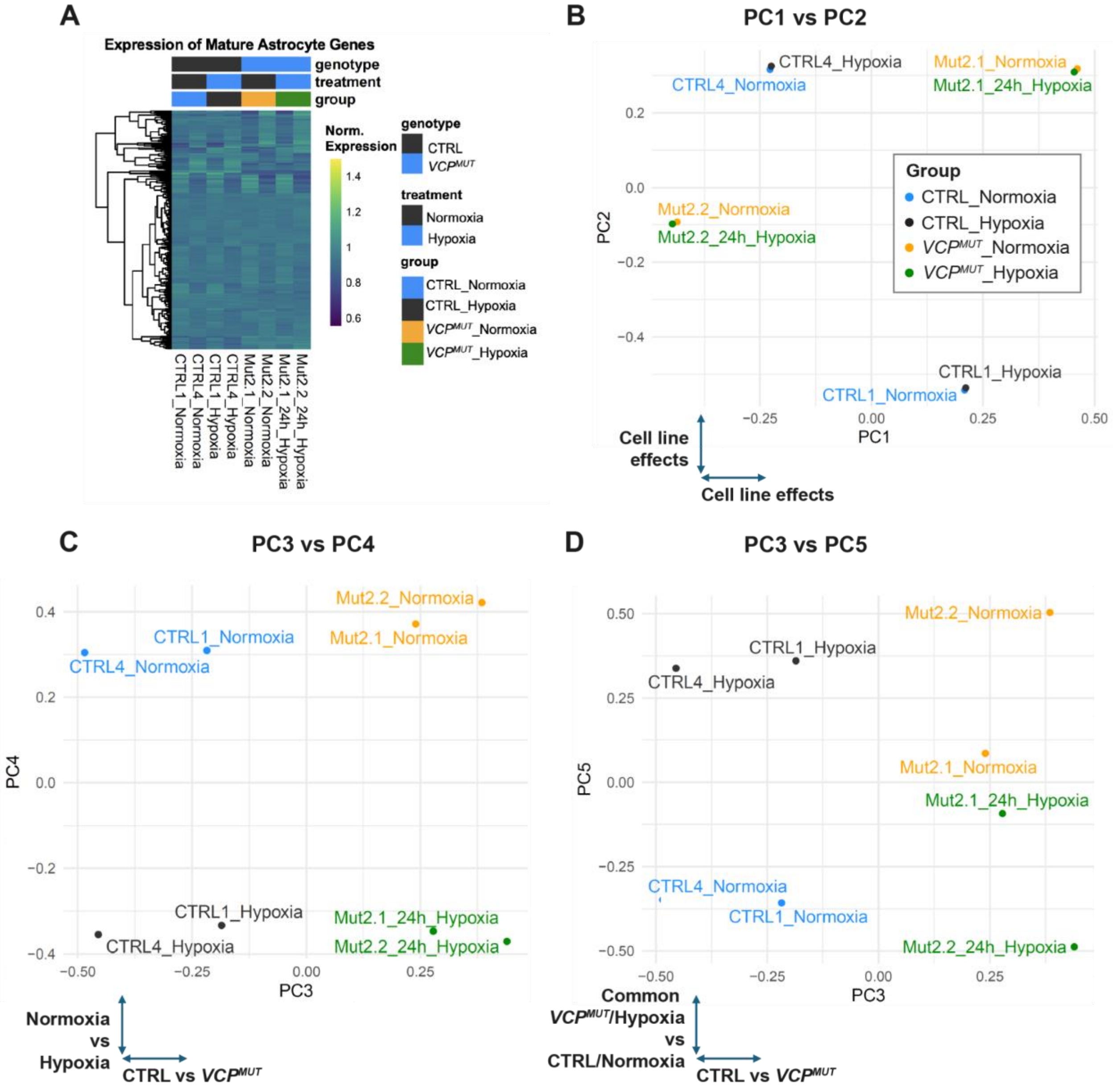

Supplementary Figure S3. Additional characterisation of transcriptomic variation in hypoxia and VCP-mutant hiPSC-derived astrocytes. **(A)** Heatmap showing bulk RNA-seq scaled gene expression for a curated panel of mature astrocyte markers. Human orthologues of mouse astrocyte maturation genes were taken from Latte et al., 2021 and mapped to our dataset. Columns are individual samples (CTRL1, CTRL4, Mut2.1, Mut 2.2) profiled under normoxia or after 24-hours hypoxia (1% O2). Rows represent genes; values are row-wise normalised (z-scored) variance stablilised counts. Unsupervised hierarchical clustering was applied to genes and samples. **(B-D)** Principal component analysis (PCA) of the top 1000 most variable genes in

hiPSC-derived astrocytes across genotype (CTRL, *VCP*^{MUT}) and oxygen condition (normoxia, hypoxia). PC1 and PC2 primarily capture variance due to cell line background. PC3 separates CTRL from *VCP*^{MUT} astrocytes, while PC4 separates normoxia from hypoxia samples. PC5 captures common variance between hypoxia-treated and *VCP*^{MUT} astrocytes compared with CTRL normoxia.

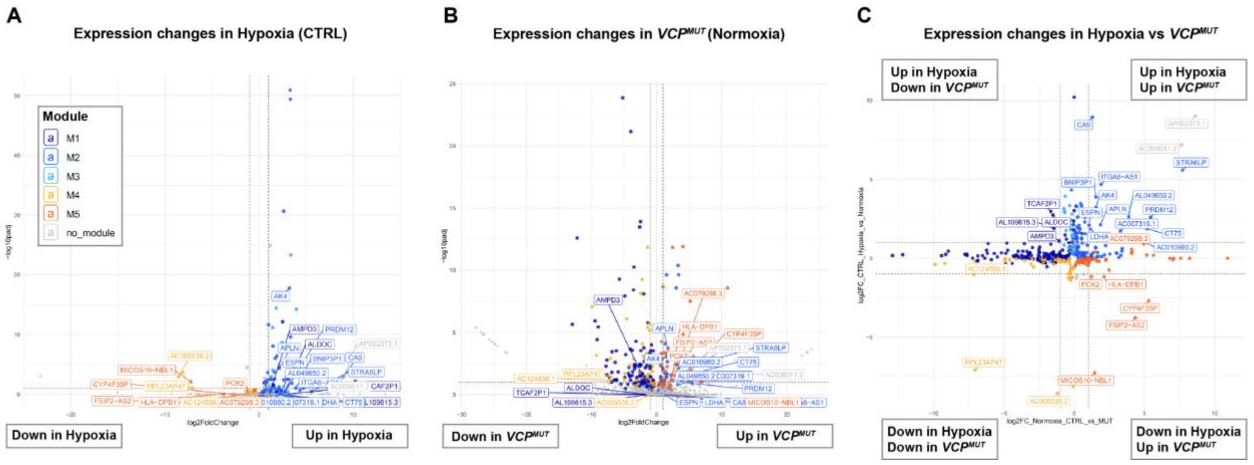

Supplementary Figure S4. Additional characterisation of differential module gene expression in hypoxia and VCP-mutant hiPSC-derived astrocytes. **(A)** Volcano plot of differentially expressed genes (DEGs) in CTRL astrocytes exposed to hypoxia vs normoxia. **(B)** Volcano plot of DEGs in *VCP*^{MUT} astrocytes versus CTRL astrocytes under normoxia. **(C)** Comparison of DEG log2 fold-changes in hypoxia versus *VCP*^{MUT} astrocytes. Genes are colour-coded by module membership (M1-M5; see Figure 2), and those with >2-fold change are labelled.

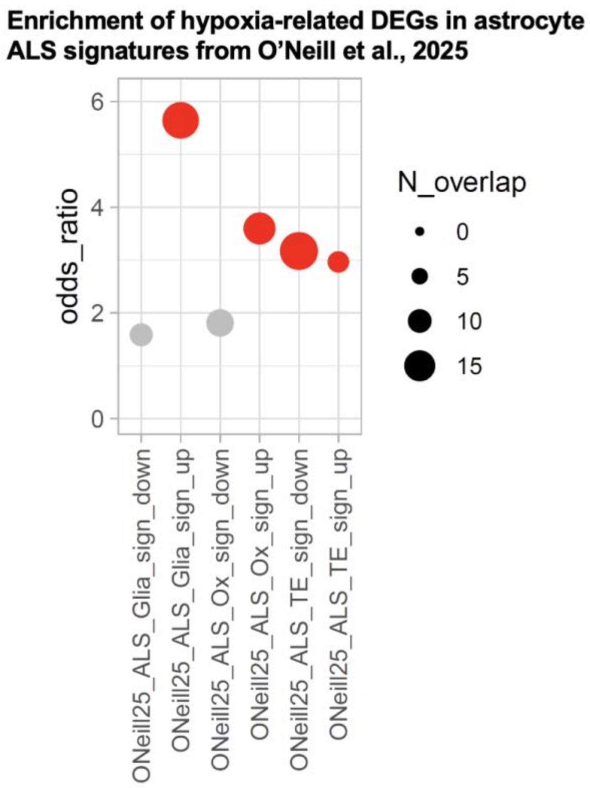

Supplementary Figure S5. Overlap of differentially expressed genes with ALS astrocyte signatures from O'Neill et al., 2025. Bubble plot showing overlap between differentially expressed hypoxia-response genes identified in this study and ALS astrocyte transcriptional signatures defined by O'Neill et al. (2025). These ALS subclasses capture mitochondrial dysfunction/oxidative stress (ALS_Ox), microglial activation/neuroinflammation (ALS_Glia), and TDP-43 pathology/transposable elements (ALS_TE). The y-axis shows the odds ratio of enrichment, while bubble size indicates the number of overlapping genes (N_overlap).

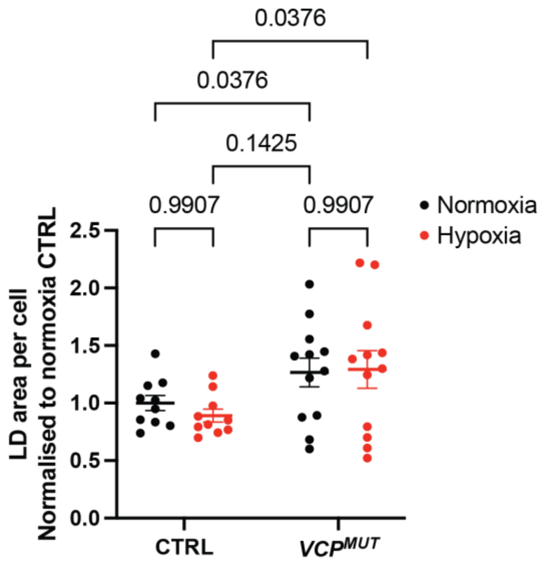

Supplementary Figure S6. Hypoxic stress has no effect on lipid droplet area in control or VCP-mutant astrocytes. Quantification of the area (pixels) of Nile Red-stained LDs per cell in CTRL and *VCP*^{MUT} astrocytes maintained under normoxia and after 24-hour exposure to hypoxia. Each data point represents the mean value of 10 fields per technical repeat per cell line (2 technical repeats per condition). Data normalised to CTRL normoxia within independent experimental repeats (cell lines used in Repeat 1: CTRL1, CTRL2, CTRL6, NCRM C2, NCRM E6, Mut1.1, Mut2.1, Repeat 2: CTRL5, CTRL6, Mut1.2, Mut2.1). *P*-values calculated from two-way ANOVA with Tukey's test for multiple comparisons. Error bars represent mean ± SEM.

Supplementary Table S1. RNA-seq module gene list, related to Figure 2.

Supplementary Methods

Derivation of human fibroblasts and human induced pluripotent stem cell (hiPSC) generation

HiPSC lines included 7 control lines (CTRL1-6, and one where we corrected the R155C mutant line to R155R) and 4 ALS *VCP*-mutant lines, including 2 clones of R155C from one patient and 2 clones of R191Q from another patient. Furthermore, we included 2 additional isogenic *VCP*-mutant lines generated through knock-in of the R191Q mutation. 3 of the control lines are commercially available and were purchased from Coriell (ND41866*C), ThermoFisher Scientific (A18945) and Cedars Sinai (CS02iCTR-NTn4). CTRL1 and the ALS *VCP*-mutant lines were kindly donated by Professor Selina Wray and her lab. They collected patient dermal fibroblasts and cultured them in OptiMEM + 10% FCS medium. For hiPSC generation, transfection of the following episomal plasmids was performed: pCXLE hOct4 shp53, pCXLE hSK, and pCXLE hUL (Addgene) (Okita et al., 2011). Details for all hiPSC lines utilised in this paper can be found in **Supplementary Table S2**.

hiPSC maintenance

Using established reprogramming methods (Okita et al., 2011), all hiPSC lines were used for directed differentiation and maintained in feeder-free, chemically defined monolayers on 6-well plates coated with Geltrex (ThermoFisher) basement membrane matrix (150 µg/ml) in Essential 8 Medium (E8) (ThermoFisher) in a humidified incubator at 37°C, 5% CO_2 and 21% O_2. Cells were fed daily with E8 and passaged at ~70% confluency using 0.5 µM Ethylenediaminetetraacetic acid (EDTA) in Dulbecco's PBS (ThermoFisher). When prepared for freezing, cells were dissociated with EDTA, transferred into a cryovial in 90% E8 media and 10% dimethylsulfoxide (DMSO) and placed in a cryopreservation container to control the rate of cooling before storing at -80°C overnight and in liquid nitrogen for long term storage. Cells were partially thawed by incubating cryovials at 37°C until few ice crystals remained, then 5 ml of E8 media was added at room temperature to complete thawing before pelleting by centrifugation at 280xg for 3 minutes. Pellets were resuspended in E8 media with 10 µM of ROCK inhibitor (Y-27632) for plating. ROCK inhibitor was removed the following day.

hiPSC-derived neural precursor generation

For differentiation to neuroepithelium, E8 media was switched to neural induction media containing a 1:1 ratio of maintenance media: N2 (DMEM/F12 Glutamax (ThermoFisher) and N2

supplement (ThermoFisher)) and B27 (Neurobasal (ThermoFisher) and B27 supplement (ThermoFisher)) with MEM NEAA (ThermoFisher), 50 U/ml penicillin-streptomycin (ThermoFisher), 5 µg/ml insulin (Sigma-Aldrich), 1 mM L-glutamine (ThermoFisher) and supplemented with 1 µM dorsomorphin (Tocris), 2 µM SB431542 (Tocris Bioscience) and 3.3 µM CHIR99021 (Miltenyi Biotec) for 7 days. At day 5, the neuroepithelial layer was enzymatically dissociated using 1 mg/ml dispase (GIBCO), washed 3 times with PBS and split 1:2 before plating onto Geltrex-coated 6-well plates. To caudalise and ventralise cells to the pMN domain of the spinal cord, neural induction media was replaced with patterning media, consisting of maintenance media supplemented with 0.5 µM retinoic acid (Sigma-Aldrich) and 1 µM purmorphamine (Merck Millipore) for a further 7 days before a 4-day phase in maintenance media and reduced purmorphamine (0.1 µM) only. During this phase, cells were expanded using 1 mg/ml dispase if necessary. After patterning and prior to terminal differentiation, neural precursor cells (NPCs) were either expanded to increase cell material by using maintenance media supplemented with 10 ng/µl FGF-2 (Peprotech) for up to 30 days, cryopreserved in DMSO for use in future experiments, or subjected to terminal differentiation. During this stage, cells were split using EDTA and plated onto Geltrex-coated plates.

Astrocyte differentiation from hiPSC-derived NPCs

For differentiation to astrocytes, NPCs were propagated further in maintenance media with 10 ng/ml FGF-2 (Peprotech) for 60-120 days to generate glial precursor cells (GPCs). Cells were split using Accutase and maintained on Geltrex-coated 6-well plates or T25/T75 flasks. Terminal differentiation was achieved with maintenance media and 10 ng/ml bone morphogenetic protein 4 (BMP4) (R&D) and 10 ng/ml leukaemia inhibitory factor (LIF) (Sigma-Aldrich) for 21 days followed by 7 days in maintenance media only. The latter is an adaptation to our original protocol (Hall et al. 2017), where differentiation for 28 days was undertaken in BMP4 and LIF. In this revised version, astrocytes are instead given 7 days without BMP4 and LIF, allowing them time to resume a more unstimulated state prior to experimentation. For final plating, cells were dissociated with Accutase and counted before plating in maintenance media into required formats on Geltrex-coated plates. Unless otherwise indicated in specific experimental procedures, the following cell counts were used: 20k cells were plated per well in 96-well plates, 120k per well in 24-well plates, 220k in 12-well plates and 500k cells per well of a 6-well plate.

Motor neuron differentiation and plating

Directed differentiation into MNs was carried out as per protocol outlined by Hall et al (Hall et al. 2017). For differentiation to neuroepithelium, E8 media was switched to neural induction media containing a 1:1 ratio of maintenance media: N2 (DMEM/F12 Glutamax (ThermoFisher) and N2 supplement (ThermoFisher)) and B27 (Neurobasal (ThermoFisher) and B27 supplement (ThermoFisher)) with MEM NEAA (ThermoFisher), 50 U/ml penicillin-streptomycin (ThermoFisher), 5 µg/ml insulin (Sigma-Aldrich), 1 mM L-glutamine (ThermoFisher) and supplemented with 1 µM dorsomorphin (Tocris), 2 µM SB431542 (Tocris Bioscience) and 3.3 µM CHIR99021 (Miltenyi Biotec) for 7 days. At day 5, the neuroepithelial layer was enzymatically dissociated using 1 mg/ml dispase (GIBCO), washed 3 times with PBS and split 1:2 before plating onto Geltrex-coated 6-well plates. To caudalise and ventralise cells to the pMN domain of the spinal cord, neural induction media was replaced with patterning media, consisting of maintenance media supplemented with 0.5 µM retinoic acid (Sigma-Aldrich) and 1 µM purmorphamine (Merck Millipore) for a further 7 days before a 4-day phase in maintenance media and reduced purmorphamine (0.1 µM) only. During this phase, cells were expanded using 1 mg/ml dispase if necessary. After patterning and prior to terminal differentiation, neural precursor cells (NPCs) were either expanded to increase cell material by using maintenance media supplemented with 10 ng/µl FGF-2 (Peprotech) for up to 30 days, snap-frozen for use in future experiments, or subjected to terminal differentiation. During this stage, cells were split using EDTA and plated onto Geltrex-coated plates. For final plating, NPCs were dissociated with Accutase (ThermoFisher) and plated into different formats on Polyethylenimine (PEI) and Geltrex-coated plates. PEI (Sigma-Aldrich) (2.2 mg/ml) was made up in 0.1 M of sodium borate (Sigma-Aldrich) and after 1-hour coating incubation at 37°C, plates were washed 3 times with tissue culture grade, sterile H2O. Following drying, plates were subsequently coated with Geltrex for 1 hour at 37°C. NPCs were counted and plated in maintenance media with 10 ng/µl FGF-2 and supplemented with 10 µM of ROCK inhibitor (Y-27632). The following day, NPCs were terminally differentiated in maintenance media and 0.1 µM Compound E (Enzo Life Sciences) to promote cell cycle exit and generate synchronised, terminally differentiated, and post-mitotic MNs. 30k NPCs were plated per well of 96-well plates.

Astrocyte-conditioned media (ACM) preparation

Spent astrocyte conditioned media (ACM) was collected per cell line after 24-hour incubation under either normoxia or hypoxia and immediately snap-frozen before storage at -80°C. Before use, ACM samples were thawed at RT and centrifuged at 2000 rpm for 3 minutes to remove cell

debris. Equal volumes of clean supernatant from individual CTRL lines were pooled together per condition to make CTRL ACM and CTRL HIACM (hypoxia-induced astrocyte conditioned media) respectively. ACM was added to hiPSC-derived MNs on day 7 of the established differentiation protocol, unless otherwise specified, in a 70:30 ratio with fresh maintenance media supplemented with Compound E (1:10000).

Hypoxia treatment

Cells were either maintained at 37°C, 5% CO_2 and 21% O_2 (normoxia), or at 1% O_2 (hypoxia). The hypoxic environment was created by use of a SCI-tive hypoxia workstation (Ruskinn Technology). All cells underwent a fresh media change prior to hypoxia treatment and all experimental manipulations were performed inside the workstation to avoid capturing effects of reoxygenation. When possible, cells were also fixed or harvested whilst still within the hypoxic environment. All cells were cultured for the same period of time, with plates being transferred from a humidified incubator, thus normoxia (21% O_2) to the hypoxia chamber (1% O_2) for the desired duration with all plates (including those kept in normoxia) being collected at the same time endpoint. Hypoxia mimetic DMOG (Sigma-Aldrich) was reconstituted in H_2O at a concentration of 30 mg/ml and added to maintenance media immediately prior to treatment with a final concentration of 500 μM.

Immunocytochemistry

Astrocytes were plated onto Geltrex-coated clear bottom 96-well plates (Falcon). At the indicated time point, media was removed and cells were washed once in PBS prior to fixing in 4% paraformaldehyde in PBS for 10 minutes at room temperature (RT). Cells were washed twice in PBS and either stored in PBS at 4°C or were permeabilised and non-specific antibody binding was blocked using 5% Bovine Serum Albumin (BSA) (Sigma-Aldrich, A7030) diluted in PBS containing 0.3% Triton X-100 (PBSTx) for 60 minutes at RT. Primary antibodies were prepared in 5% BSA/PBSTx at the required dilution (see Supplementary Table S3) and applied to cells for 1 hour at RT, or overnight at 4°C. Cells were washed twice in PBS before primary antibody detection using InvitrogenTM Alexa FluorTM secondary antibodies (1:1000) and DAPI nuclear stain (1:2000 in 5% BSA/PBSTx) for 1 hour in the dark at RT. Secondary antibodies and DAPI were removed using 2 further PBS washes. Cells on glass coverslips were mounted onto slides using Dako Fluorescence Mounting Medium (Agilent) and cells in 96-well plates were stored in PBS. Cells were then imaged as detailed below.

High throughput confocal imaging and analysis

HiPSC-derived astrocytes plated in 96-well formats were visualised using the Perkin Elmer Opera Phenix™ High Content Screening System with a 40x water objective. For each well a minimum of 8 fields were acquired. Z stacks of images were acquired with a minimum of 5 slices per stack, with images displayed as maximum projections. Acquisition and thresholding settings were standardised for each experimental block. Cells were analysed with the complementary Columbus™ Image Data Storage and Analysis system.

Real-time quantitative polymerase chain reaction (RT-qPCR)

Astrocytes were plated onto Geltrex-coated 12- or 24-well plates. RNA was extracted using a Maxwell® RSC simplyRNA Cells Kit (Promega, AS1390) and a Maxwell® RSC 48 Instrument. At the indicated time point, media was removed, and cells were washed once with PBS before homogenisation in 200 µl of chilled 1-Thioglycerol/homogenisation solution and storage on ice followed by adding 200 µl of lysis buffer and vortexing for 15 seconds. Lysed samples were then added to Maxwell® cartridges with 5 µl DNase and eluted in 35 µl nuclease-free water. RNA quality and concentration was quantified using a NanodropTM 2000/2000c Spectrophotometer (Thermo Scientific) and stored at -80°C.

200 ng of RNA was added for reverse transcription into cDNA using the RevertAid First Strand cDNA Synthesis Kit (ThermoFisher, K1621). RNA was added into a sterile nuclease-free tube on ice with 1 µl Random Hexamer primer and made up to 12 µl total volume with nuclease-free water. Each reaction was then gently mixed and spun down before incubating at 65°C for 5 minutes. The following reagents were then added to each reaction on ice: 4 µl Reaction Buffer, 1 µl RiboLock RNase Inhibitor, 2 µl 10mM dNTP Mix and 1 µl RevertAid M-MuLV RT for a total volume of 20 µl. Reactions were gently mixed and incubated at 25°C for 5 minutes, followed by 4°C for 60 minutes before reaction termination at 70°C for 5 minutes. Samples were run alongside a negative control in the absence of RevertAid M-MuLV RT and stored at -20°C.

cDNA was diluted 1:20 before qPCR amplification, which was carried out using PowerUp SYBR Green Master Mix (ThermoFisher) on a QuantStudio 6 Flex Real-Time PCR System (Applied Biosystems). All qPCR primers (listed in **Supplementary Table S4**) were designed using NCBI

primer blast software and subjected to quality control validation using melt curve analysis and amplification characteristics. Primer pairs with efficiencies between 90-110% were used and RT-negative controls were used in all experiments. Gene expression levels were normalised over the housekeeping gene *ACTB*.

Western blot

Western blotting was performed according to standard protocols (BioRad). Whole cell lysates were obtained by washing cells with ice cold PBS before adding RIPA lysis and extraction buffer (ThermoFisher) to wells containing astrocytes post-treatment. Protein concentration was quantified using Pierce BCA assay (ThermoFisher) to maximise even loading between samples (~30 μg per sample/lane). Electrophoresis was run on NuPAGETM 4-12% Bis-Tris 1.0mm 10-well gel (Invitrogen) with 1X MOPS SDS running buffer (Invitrogen) at constant voltage (160V) for 75 minutes. Samples were run alongside PageRulerTM Plus prestained protein ladder (ThermoFisher Scientific). Electrophoresis was followed by protein transfer to a nitrocellulose membrane (BioRad) with NuPAGETM transfer buffer (Invitrogen). Blocking was performed in 5% dry milk powder in PBS - 0.1% Tween (PBS-T) at RT for 1 hour, followed by sequential primary antibody incubation at 4°C overnight. Primary antibodies were diluted in 5% dry milk powder/PBS-T as follows: mouse anti-human HIF-1α (BD Biosciences, 610959) 1:500, mouse anti-beta Actin (GeneTex, GT5512) 1:10000. For detection of primary antibodies, membranes were incubated with anti-mouse infra-red fluorescent antibodies (IRdye, Licor) for 1 hour at RT and imaged using an Odyssey Fc Imaging System (Licor). Results were quantified using Image StudioTM Lite software (Licor).

Live-cell quantification of mitochondrial membrane potential

Mitochondrial function was indicated by measurement of the mitochondrial membrane potential (MMP) using the cationic dye tetramethylrhodamine methyl ester (TMRM). Due to its positive charge, TMRM accumulates within the mitochondria in an inverse proportion to MMP. While these dyes can be used in a “quenching” mode at high concentrations between 1 and 20 μM, they can also be used in non-quenching mode at much lower concentrations to ensure mitochondrial function is not altered by the presence of the dye.

For the purpose of this assay, hiPSC-derived astrocytes were plated in 96-well formats under normoxic or hypoxic conditions in the absence or presence of drug treatments for 24 hours. A 50 μM stock solution of TMRM (Invitrogen) in DMSO was diluted in maintenance media to make a

relative working concentration of 60 nM per well. 50 µl was then added to 100 µl media (or ACM in the case of non-cell autonomous experiments) already in each well to make a final concentration of 20 nM per well, before a 45-minute incubation period in the dark at 37°C. After 35 minutes, 10 µl of 10 mg/ml Hoechst 33342 (Thermo) nuclear stain diluted in maintenance media (1:2000) was added to each well for 10 minutes in the dark. All media/dye conditions were then replaced with fresh maintenance media for live-cell imaging. Hypoxic cells were maintained in hypoxic conditions throughout all media changes until immediately prior to imaging.

Cells were visualised using the Perkin Elmer Opera Phenix™ High Content Screening System and analysed with the complementary Columbus™ Image Data Storage and Analysis system. Nuclei were defined using the Hoechst stain channel and border objects were excluded. To identify astrocytes selectively, nuclear size and Hoechst intensity parameters were used to exclude pyknotic and non-astrocytic cells. A ring region was then defined to represent the cytoplasmic region. Using these masks, average intensities were then measured for each individual cell. Average intensity values per well were used to represent the final experimental outcome.

Mitochondrial area measurement

Mitochondria were labelled using MitoTracker™ Green FM (ThermoFisher). Immediately prior to use, the vial was equilibrated to RT and dissolved in 74.42 µl DMSO to prepare a 1 mM stock solution. A working solution was then prepared at 3.4x the desired final concentration, such that 50 µl was added per well of a 96-well plate containing 120 µl medium, yielding a final concentration of 100 nM MitoTracker. Hoechst 33342 (1:2000; ThermoFisher) was included to label nuclei. Cells were incubated with the staining solution for 30 minutes at 37°C protected from light, washed once with PBS, and returned to maintenance medium for immediate live-cell imaging on the Opera Phenix Plus High-Content Screening System (PerkinElmer). Image analysis was performed using the complementary Columbus™ Image Data Storage and Analysis system, measuring mitochondrial area per cell.

Lipid droplet measurement

Detection and quantification of intracellular lipid droplets was achieved using lipophilic fluorescent dye, Nile Red (ThermoFisher). Non-fluorescent in water and most polar solvents, Nile Red undergoes intense fluorescence enhancement in nonpolar, lipid-rich, environments. For the purpose of this assay, cells were plated on 96-well plates and maintained in either normoxic or

hypoxic conditions ± drug treatments. A 1 mM dye stock solution was prepared in DMSO and kept in the dark at RT. Immediately prior to use, a working concentration of 3 μM was prepared in 50 μl maintenance media per well and added to 100 μl of maintenance media (± drug conditions) already in the well at the end of the 24-hour treatment period to make up a final concentration of 1 μM, followed by a 10-minute incubation in the dark at 37°C. Cells were then washed once in PBS, before 10-minute fixation in 4% PFA. Cells were washed once more in PBS, immunolabelled with GFAP and stained with DAPI.

Cells were then visualised using the Perkin Elmer Opera Phenix™ High Content Screening System and analysed with the complementary Columbus™ Image Data Storage and Analysis system. Nuclei were defined using the DAPI stain and border objects were excluded. To select astrocyte populations, nuclear size and DAPI intensity parameters were used to exclude pyknotic and non-astrocytic cells. Using GFAP, a cytoplasmic mask was generated and using the integrated "Find Spots" function, Nile Red puncta located within the cytoplasmic region were identified. The number of spots and spot area were calculated per cell and averaged per well.

Intracellular ROS measurement

Oxidative stress was measured by detection of intracellular ROS using a fluorogenic probe, CellROX™ Green (ThermoFisher). CellROX™ Green displays low fluorescence within intracellular environments in a reduced state. Upon oxidation by ROS and subsequent binding to DNA, the dye exhibits robust green photostable nuclear fluorescence. For the purpose of this assay, cells were plated on Geltrex-coated 96-well plates and maintained under either normoxic or hypoxic conditions ± drug treatments. The 2.5 mM dye stock solution was prepared in 30 μl maintenance media per well and added to 100 μl of maintenance media (± drug conditions) already in the well at the end of a 24-hour treatment period to make up a final concentration of 5 μM, followed by a 30-minute incubation in the dark at 37°C. Cells were then washed once with PBS, before 10-minute fixation in 4% PFA and a further two PBS washes. Hypoxic conditions were maintained for the duration of dye incubation period, fixation and washes. Fixed and stained astrocytes were then visualised using the Perkin Elmer Opera Phenix™ High Content Screening System and analysed with the complementary Columbus™ Image Data Storage and Analysis system, with intracellular ROS being quantified by nuclear intensity of CellROX™ Green.

RNA sequencing sample preparation

Poly(A)+selected reverse stranded RNA sequencing libraries were prepared from 2 control and 2 *VCP*-mutant lines, under basal conditions (normoxia) or after exposure to 24-hour 1% O2 hypoxia, using the KAPA mRNA HyperPrep Library kit for Illumina, with 50 ng of total RNA as input. Libraries were sequenced on the NovaSeq 6000 platform.

Cleavage Under Targets and Release Using Nuclease (CUT&RUN) sample preparation

hiPSC-derived astrocytes were left untreated or treated with 500 µM DMOG for 24 hours. Cells were fixed directly in the wells for 2 minutes at 37ºC by adding PFA to a final concentration of 0.1% directly to growth media. Fixation was then quenched by the addition of 125 mM glycine for 5 minutes at 37ºC. Cells were collected in single cell suspension and then counted to ensure equal numbers were loaded into each reaction. Samples were processed using the Cell Signalling CUT&RUN assay kit (#86652) according to the manufacturer's instructions. Briefly; cells were washed twice in wash buffer containing protease inhibitor cocktail and spermidine before resuspension in wash buffer. 10 µl of concalvin A magnetic beads were washed and activated per reaction before binding to cells. Cells and beads were incubated overnight rotating at 4ºC in antibody buffer containing digitonin (activated at 95ºC for 5 minutes), protease inhibitor cocktail, spermidine and 0.5 µg of HIF-1α antibody (Novus Biologicals # NB100-134). Following incubation, samples were washed in wash buffer containing digitonin (activated at 95ºC for 5 minutes), protease inhibitor cocktail and spermidine, before adding pAG-MNase to samples incubated for 1 hour at 4ºC, followed by further washes. $CaCl_2$ was then added for activation, followed by 30 minutes incubation at 4ºC. The reaction was terminated at 37ºC for 10 minutes by addition of Stop buffer containing digitonin, RNAse A and E. coli DNA spike in. Cross linking was then reversed by overnight incubation at 65ºC with SDS and Proteinase K before purifying samples using Zymo Research DNA clean & concentrator columns.

Sequencing libraries were prepared using the Cell Signaling DNA library Prep kit for Illumina sequencing (#56795) and the Multiplex Oligos for Illumina Systems (Single Index Primers) (#29580) according to the manufacturer's instructions. Briefly; End Prep enzyme mix and buffer were added directly to samples followed by incubation at 20ºC for 30 minutes then 50ºC for 30 minutes. Adapters were diluted 1:50 in 10 mM Tris before addition to samples, followed by incubation for 15 minutes at 22ºC before USER enzyme was added and incubated at 37ºC for 15 minutes to complete ligation. Prior to PCR enrichment and primer addition, samples were purified using MagSafe beads. Cycling conditions for library amplification and indexing primer addition

were as follows; 98ºC for 30 minutes, 15 cycles of 98ºC for 10 minutes, 65ºC for 13 minutes, and 65ºC for 3 minutes. Samples were purified by two rounds of Magsafe bead clean up. Libraries were analysed by Tapestation and diluted to 4 nM pools before sequencing using the NovaSeq 6000 Sequencing technology at 8 million 100bp paired end reads per sample.

CUT&RUN bioinformatic analysis

CUT&RUN fastq files were analysed using the nf-core/cutandrun pipeline (v3.2.2, doi:10.5281/zenodo.10606804). In this pipeline, reads were mapped to the human genome GRCh38 (hg38) using Bowtie2 (Langmead and Salzberg 2012), with the following settings: --end-to-end --very-sensitive --no-mixed --no-discordant -q --phred33 -I 10 -X 700. We used Picard (McKenna et al., 2010) to mark duplicate reads, and SAMtools (Li et al. 2009) was used to convert and index SAM files into BAM files. Reads were also aligned to the E. coli K12-MG1655 reference genome and spike-in normalisation was performed using BEDtools (Quinlan and Hall 2010). SEACR (Meers, Tenenbaum, and Henikoff 2019) was used to call peaks with default parameters. Fragment- and peak-based quality control checks were performed using deepTools (“Website,” n.d.-a). Differentially expressed genes were considered potential HIF1A targets when at least one peak binding *HIF1A* in the CTRL_DMOG condition was assigned to the respective gene. Peaks were annotated using the R Package ChiPseeker (“Website,” n.d.-b) with the transcript database TxDb.Hsapiens.UCSC.hg38.knownGene as the input.

For each sample, peak calling was performed using SEACR (Sparse Enrichment Analysis for CUT&RUN) with the parameter setting “0.005 non stringent output”. This approach identifies candidate enriched regions by applying a stringent threshold on signal profiles. Further, this allowed only the top 0.5% of regions ranked by Area Under the Curve (AUC) values, thereby restricting downstream analyses to the most highly enriched genomic intervals. Further, we overlapped these peaks with H3K4me3 peaks using the bedtools intersect command to confirm that the identified peaks were highly specific to promoter regions, as would be expected from *HIF1A* binding.

Analysis and integration of RNAseq and CUT&RUN

RNA-seq reads from fastq files were mapped to the human genome (GRCh38) using the nf-core/rnaseq nextflow pipeline (v3.5, doi:10.5281/zenodo.1400710). Gene level reads were extracted in R (v4.4.0). After removing lowly expressed genes (<= 0.5 counts per million), differential expression analysis was performed with DESeq2 (v1.46.0) (Love, 2014), using a

model accounting for genotype and treatment with the commands DESeqDataSetFromMatrix(... design = ~treatment*genotype) and DESeq(dds, test = "LRT", reduced = ~1), or, for pairwise comparisons (Supplementary Figure S4), using DESeqDataSetFromMatrix(..., design = ~group). Differentially expressed genes (FDR <= 0.05) were then grouped into co-expressed modules using the function degPatterns(minc = 10, time = "treatment", col = "genotype") from the Bioconductor R package DEGreport (v1.42.0, DOI: 10.18129/B9.bioc.DEGreport) on the vst-normalised expression matrix. Functional enrichment analyses for Gene Ontology terms and gene sets from the Molecular Signatures Database (MSigDB) was performed using the Bioconductor R packages clusterProfiler (v4.14.1) with org.Hs.eg.db (v3.20.0), DOSE (v4.0.0) and msigdbr (v7.5.1). The standard R packages ggplot2 (v3.5.1) and pheatmap (v1.0.12) were used for visualisations. As a broad signature of canonical hypoxia-regulated genes, we used genes occurring in any of the following hypoxia gene sets from the MSigDB - "HALLMARK_HYPOXIA", "GOBP_RESPONSE_TO_OXYGEN_LEVELS", “QI_HYPOXIA", "HARRIS_HYPOXIA", "LEONARD_HYPOXIA", "KIM_HYPOXIA".

Supplementary Table S2. HiPSC lines used in study

**HiPSC cell line**	***VCP* mutation loci**	**Age of donor**	**Age at disease onset**	**Sex of donor**	**Source**
CTRL1	-	78	-	Male	Wray Lab
CTRL2	-	64	-	Male	Coriell (ND41866*C)
CTRL3	-	Foetal	-	Female	ThermoFisher Scientific (A18945)
CTRL4	-	51	-	Female	Luke Hill (Patani Lab)
CTRL5	-	51	-	Male	Cedars Sinai (CS02iCTR-NTn4)
CTRL6 (NCRM1)	-	Foetal	-	Male	NIH-CRM (CRMi003-A)
VCPF10	(Corrected R155R from mutant R155C)	43	40	Female	Wray Lab
NCRM C2	R191Q (isogenic inserted)	Foetal	-	Male	CRMi003-A - edited by Synthego
NCRM E6	R191Q (isogenic inserted)	Foetal	-	Male	CRMi003-A - edited by Synthego
Mut1.1	R191Q	42	36	Male	Wray Lab
Mut1.2	R191Q	42	36	Male	Wray Lab
Mut2.1	R155C	43	40	Female	Wray Lab

Mut2.2	R155C	43	40	Female	Wray Lab

Supplementary Table S3. Primary antibodies used for immunofluorescence in this study

Primary Antibody	Species	Dilution	Manufacturer
βIII-tubulin	Chicken	1:1000	Abcam (ab41489)
FUS	Mouse	1:200	Santa Cruz (sc-47711)
GFAP	Chicken	1:1000	Abcam (ab4674)
HIF-1α	Rabbit	1:200	Sigma-Aldrich (SAB2702132)
SFPQ	Mouse	1:400	Abcam (ab11825)

Supplementary Table S4. Primer sequences used for RT-qPCR in this study

Gene target	Forward sequences (5'-3')	Reverse sequences (5'-3')
HIF-1α	TTCCTTCTCTTCTCCGCGTGTGG	CTTTTTCTTGTCGTTCGCGCCG
VEGF	CCAATCGAGACCCTGGTGGACA	GGTGAGGTTTGATCCGCATAATCTGC
PDK1	GTGGATCCTGTCACCAGCCAGA	TTCCACCAAACAATAAAGAGTGCTGA
ANG	TGGCAACAAGCGCAGCATCAG	GCAAGTGGTGACCTGGAAAGAAG
ACTB	GGGGTGTTGAAGGTCTCAAA	GGCATCCTCACCCTGAAGTA
